# Global, Regional, and National Trends of Chagas Disease from 1990 to 2019: Comprehensive Analysis of the Global Burden of Disease Study

**DOI:** 10.5334/gh.1150

**Published:** 2022-08-24

**Authors:** Sergio Alejandro Gómez-Ochoa, Lyda Z. Rojas, Luis E. Echeverría, Taulant Muka, Oscar H. Franco

**Affiliations:** 1 Public Health and Epidemiological Studies Group, Cardiovascular Foundation of Colombia, Floridablanca, Colombia; 2 Research Group and Development of Nursing Knowledge (GIDCEN-FCV), Research Center, Cardiovascular Foundation of Colombia, Floridablanca, Santander, Colombia; 3 Heart Failure and Heart Transplant Clinic, Fundación Cardiovascular de Colombia, Floridablanca, Colombia; 4 Institute of Social and Preventive Medicine (ISPM), University of Bern, Bern, Switzerland

**Keywords:** Chagas Disease, Prevalence, Public Health

## Abstract

**Background:**

Chagas disease (CD) is a neglected tropical disease, endemic in Latin America, but due to migration and environmental changes it has become a global public health issue.

**Objectives:**

To assess the global prevalence and disability-adjusted life years due to CD using findings from the Global Burden of Disease Study 2019.

**Methods:**

The Global Burden of Disease data was obtained from the Global Burden of Disease Collaborative Network; results were provided by the Institute for Health Metrics and Evaluation. The prevalence and disability-adjusted life-years (DALYs) were described at a global, regional, and national level, including data from 1990 to 2019.

**Results:**

Globally, CD prevalence decreased by 11.3% during the study period, from 7,292,889 cases estimated in 1990 to 6,469,283 in 2019. Moreover, the global DALY rate of CD decreased by 23.7% during the evaluated period, from 360,872 in 1990 to 275,377 in 2019. In addition, significant differences in the burden by sex, being men the most affected, age, with the elderly having the highest burden of the disease, and sociodemographic index (SDI), with countries with the lowest SDI values having the highest prevalence of the disease, were observed. Finally, the prevalence trends have followed different patterns according to the region, with a sustained decrease in Latin America, compared to an increasing trend in North America and Europe until 2010.

**Conclusion:**

The global burden of CD has changed in recent decades, with a sustained decline in the number of cases. Although the majority of cases remain concentrated in Latin America, the increase observed in countries in North America and Europe highlights the importance of screening at-risk populations and raising awareness of this neglected tropical disease.

## Introduction

Chagas Disease (CD), also known as American trypanosomiasis, is a neglected tropical disease caused by the protozoa *Trypanosoma cruzi*, which is endemic in 21 countries of continental Latin America. CD’s geographic distribution was mostly driven by the distribution of the vector species in the Americas regions below 1500m elevation. However, due to migration, it has become a global health threat [1]. According to the World Health Organization, six to eight million people are estimated to be infected with *T. cruzi*, and another 70 million are estimated to be at risk of infection [2]. This infectious disease has two phases: acute and chronic; the latter is most commonly associated with myocardial involvement, known as Chronic Chagas Cardiomyopathy (CCM) [3]. The pathophysiology of the progressive myocardial damage remains poorly understood; however, recent evidence suggests an essential role of T. cruzi persistence (both in blood and tissues) and a pro-inflammatory immunological profile in the host, which is reflected in sustained oxidative stress, and natural killer/CD8+ T-cell cytotoxicity [4,5].

The conjunction of heart failure and systemic embolisms in Chronic Chagas Cardiomyopathy (CCM), the most severe form of organ involvement in CD, has been associated with a significant burden in the affected patients [6]. CCM has been associated with higher morbidity and mortality rates compared to other cardiomyopathies, mainly due to severe myocardial injury, which not only leads to heart failure, but also favors conduction disorders and new-onset arrhythmias such as atrial fibrillation, which, together with the presence of myocardial aneurysms, leads to a significantly increased risk of embolic events [7]. On the other hand, it is estimated that CD leads to a global expenditure of USD 627.5 million per year, mainly due to health care costs. Precisely, it is estimated that the cost per patient rises from $200 in the early stages of the disease to $6,000 in the chronic forms [8]. As a result, CD has been ranked as the parasitic disease with the highest attributable burden worldwide [9].

Although some mathematical models and systematic reviews have aimed to estimate CD’s global burden, a detailed quantitative analysis of this disease burden is unfortunately not available [10]. We aimed to characterize in detail the global state of CD epidemiology by using the Global Burden of Disease (GBD) data from 1990 to 2019, highlighting the differences by sex and the most affected ages across regions and nations.

## Methods

### Data Sources

The Global Burden of Diseases, Injuries, and Risk Factors Study (GBD) is an international initiative aimed to provide a systematic scientific evaluation of published data regarding disease and injury incidence, prevalence, and mortality for a wide list of diseases and injuries. We used the GBD 2019 results to assess the global burden of Chagas Disease (CD) by extracting data on CD prevalence, death due to CD, disability-adjusted life-years (DALYs), and their uncertainty intervals from GBD data sources. In summary, the GBD study performed an epidemiologic assessment of 333 diseases by age and sex globally for the years 1990 to 2019. The method by which the GBD estimates Chagas Disease burden in endemic countries is based on seroprevalence data with an integrative Bayesian meta-regression technique, which assesses a generalized negative binomial model for all epidemiological data through DisMod-MR 2.1. On the other hand, in non-endemic countries, the estimates are calculated by estimating the importation of prevalent cases via immigration. Data from the Pew Research Trust’s Center (https://www.pewresearch.org/hispanic/2014/04/29/2012-statistical-information-on-immigrants-in-united-states/) and the International Migrant Stock of the United Nations are used to estimate the number of foreign-born individuals by age, sex, and country of origin for each state. Finally, the state-specific estimates of the number of people born in CD-endemic countries and the estimated prevalence from each country are used to generate state-specific CD prevalence estimates.

We obtained the GBD data from the Global Burden of Disease Collaborative Network; results were provided by the Institute for Health Metrics and Evaluation (Seattle, WA). In this context, the GBD cause list is organized hierarchically into four levels, Chagas disease is in level 2 group of “Neglected tropical diseases and malaria” and level 1 group of “Communicable, maternal, neonatal, and nutritional diseases.” Furthermore, Chagas disease’s case definition was based on the following International Classification of Diseases (ICD)-9 codes: 086.0–086.2 and 425.6, and ICD-10 codes: B57-B57.5 and K93.1. For the generation of non-fatal estimates for Chagas Disease, GBD used existing data from population studies of Chagas disease seroprevalence worldwide.

We considered the following as endemic countries due to the high circulation of *T. cruzi* strains according to the available literature: Argentina, Belize, Bolivia, Brazil, Chile, Colombia, Costa Rica, Ecuador, El Salvador, French Guyana, Guatemala, Guyana, Honduras, Mexico, Nicaragua, Panama, Paraguay, Peru, Suriname, Uruguay, and Venezuela [2].

### Measures of Burden

The measures of burden assessed in the present study included prevalence, death due to CD, and DALYs due to CD. At first, DALYs were extracted directly from the GBD. This measure is calculated by adding years lived with any short- or long-term disability and years of life lost (YLL) due to premature death. The years lived with disability (YLD) is a burden measure that represents the number of years lived with CD considering the related disability weights to reflect CD’s underlying severity. Both YLL and YLD were estimated specifically by age, sex, and country. All measures were reported as number counts and age-standardized rates per 100,000 population. This age standardization was based on the World Health Organization world population standard age structure.

### Statistical Analysis

Global, regional and national data from the countries with a prevalence rate per 100.000 population higher than 0.1 was presented. Results of prevalence, deaths, DALYs, YLLs, and YLDs were presented in absolute numbers and age-standardized rates per 100,000 population, along with their respective 95% uncertainty intervals (UIs). We also reported the percentage of change in the values between 1990 and 2019 to show the variations in this period. Finally, we performed a literature review in Pubmed and LILACS to assess new primary data concerning the prevalence of CD in endemic countries to generate an updated estimate and compare it with the GBD data and other estimates published in the literature. Furthermore, we analyzed the CD prevalence rate per 100,000 population between countries according to their sociodemographic index classification (SDI), a composite indicator of development status developed by GBD researchers (https://ghdx.healthdata.org/record/ihme-data/gbd-2019-socio-demographic-index-sdi-1950-2019). The Kruskal–Wallis one-way analysis of variance test was used to compare the prevalence rate among the different SDI categories. A p-value < 0.05 was considered significant. All tests were two tailed. All statistical analyses were performed using R Statistical Software (version 4.2.1; R Foundation for Statistical Computing, Vienna, Austria).

## Results

### Overall Burden

By 1990, the global prevalence of CD was estimated in 7,292,889 (95% uncertainty interval [UI] = 6,348,544.14- 8,359,225.33), with an age-standardized prevalence rate per 100,000 population of 145.07 (95% UI = 126.74–165.59). By this year, Bolivia was the country with the highest prevalence rate per 100,000 population (14,498; 95% UI = 12,803.28–16,303.82), followed by Argentina (4,987; 95% UI = 4,423.46–5,566.96) and Chile (3,911; 95% UI = 3,485.31–4,430.37). Furthermore, Brazil was the country with the higher number of estimated CD cases with around 1,918,996.9 cases (26.3% of the total), followed by Argentina (1,622,896.4 cases, 22.3% from the total) and Mexico (939,303.44, 12.9% from the total). Moreover, the prevalence in non-endemic countries in 1990 represented only 0.86% (n = 71,372) from the total cases, highlighting Israel as the country with the highest prevalence rate per 100,000 population (40.4; 95% UI = 35.63–45.38), while in the United States (U.S.) the state of Florida was estimated to be the region with the highest prevalence rate per 100,000 population (60.7; 95% UI = 53.51–69.45). Finally, the U.S. was the non-endemic country with the largest estimated number of cases in 1990 (53,505.66; 95% UI = 46,913.63– 61,492.67).

**Figure 1:**
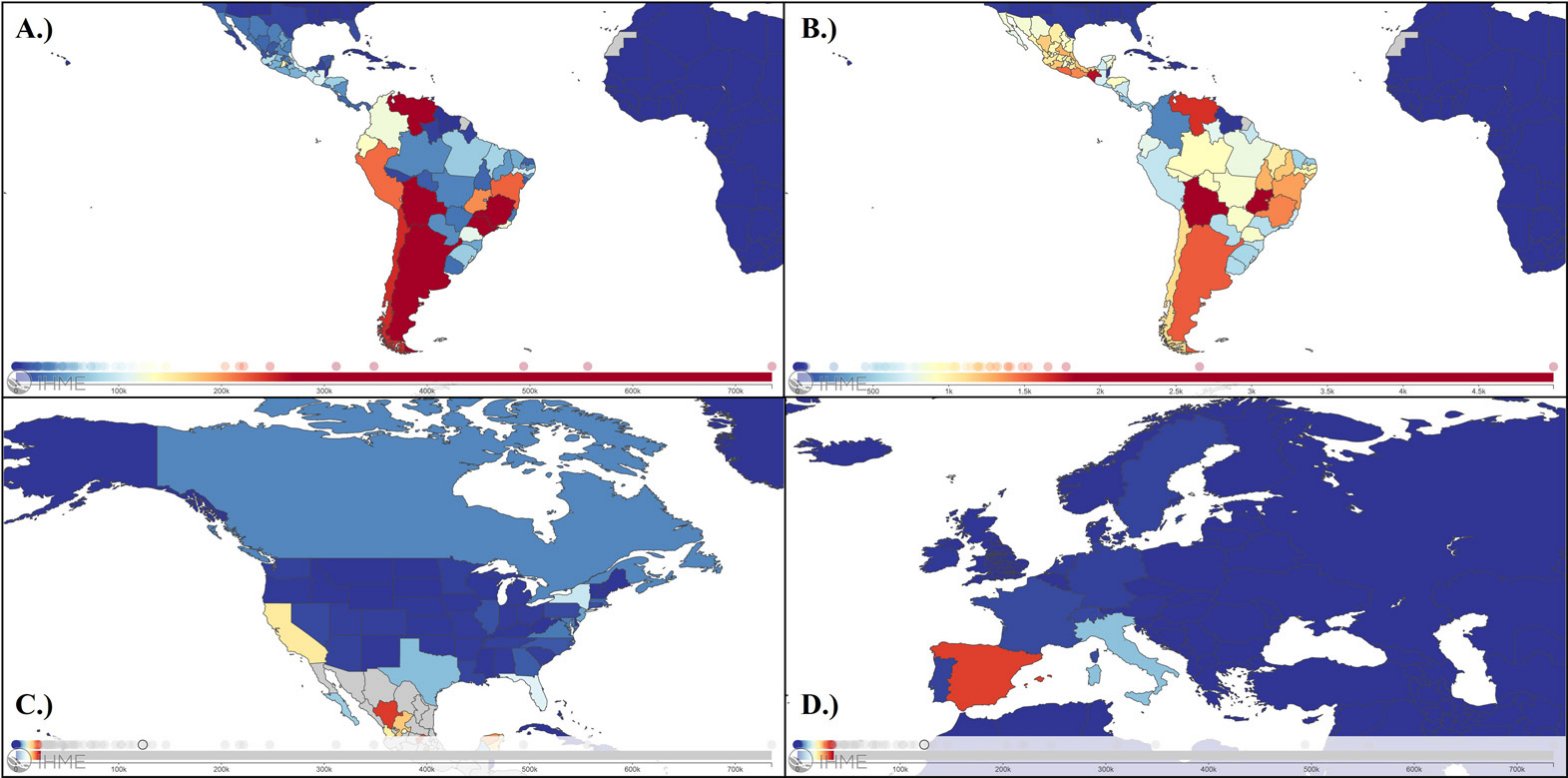
World map summarizing **A.)** the prevalence number of Chagas Disease (CD) reported cases by country in Latin America, **B.)** the age-standardized prevalence rate of CD by country in Latin America, **C.)** The prevalence number of CD reported cases by country and U.S. state in North America, and **D.)** The prevalence number of CD reported cases by country in Europe in 2019. Source: https://vizhub.healthdata.org/gbd-compare/.

During the year 2019, the global prevalence of CD was 6,469,283 (95% UI = 5,658,409.78–7,374,556.70), showing a decreasing trend compared to the prevalence reported in 1990. This difference represented a 11.3% decrease in prevalence over the last three decades. Similarly, the age-standardized prevalence rate per 100,000 population in 2019 decreased to 83.61 (95% UI = 73.13–95.31) representing a 42% reduction of this value with respect to 1990 (Supplementary Figure 1). Bolivia remained the country with the highest prevalence rate per 100,000 population (4993.53; 95% UI = 4540.19 – 5483.11), followed by Venezuela (1654,73; 95% UI = 1439.50– 1897.81) and Argentina (1524.07; 95% UI = 1343.16 – 1736.00) (Supplementary Figure 2). On the other hand, Brazil remained the country with the higher number of estimated CD cases, with around 2,164,570 cases (33.5% of the total). Furthermore, Argentina was the endemic country with the largest reduction in the prevalence number (–54.68%), followed Chile (–50.95%) and Uruguay (–49.95%), while Mexico (39.58%), Brazil (12.80%) and Honduras (14.12%) were the endemic countries with the largest increase in estimated number of cases in the period between 1990 and 2019. Conversely, none of the endemic countries had an increase in the prevalence rate per 100,000 population. On the other hand, the estimates suggest that Spain was the country with the largest absolute increase in the number of estimated CD cases (25,081 cases), followed by the United States of America (10,048 cases) and Italy (5542 cases). Finally, the U.S.A remained the non-endemic country with the largest estimated number of cases by 2019 (63,553.17; 95% UI = 55,365.83–72,997.92), being California the state with the highest prevalence number (13,600; 95% UI = 11,761.33–15730.08). Table 1 and Figure 1 present a summary of the prevalence data by country and U.S.A states.

**Table 1:** Top 10 countries with the highest estimated Chagas Disease prevalence rate per 100,000 population in 2019.


COUNTRY/STATE	PREVALENCE (RATE PER 100,000 POPULATION) 1990	PREVALENCE (RATE PER 100,000 POPULATION) 2019	PERCENTAGE CHANGE 1990–2019 (%)	PREVALENCE NUMBER 1990	PREVALENCE NUMBER 2019	PERCENTAGE CHANGE 1990–2019 (%)

***Bolivia (Plurinational State of)***	14498.60 (12803.28–16303.82)	4993.53 (4540.19–5483.11)	–65.56	795786.94 (700556.5–899392.56)	556181.13 (507218.66–611029.13)	–30.11

***Venezuela (Bolivarian Republic of)***	2888.71 (2495.06–3327.43)	1654.73 (1439.50–1897.81)	–42.72	457479.5 (392331.28– 530739.19)	493902.31 (429738.44– 564220)	7.96

***Argentina***	4987.04 (4423.46– 5566.96)	1524.07 (1343.16– 1736.00)	–69.44	1622896.4 (1441816.8 – 1811864.5)	735490.88 (648796.81– 838063)	–54.68

***Chile***	3911.59 (3485.31– 4430.37)	1114.17 (987.04– 1261.69)	–71.52	503984.09 (448713.91– 571287.44)	247197.23 (219291.86– 279687.94)	–50.95

***Mexico***	1347.48 (1120.54– 1614.53)	1030.50 (877.70– 1205.66)	–23.52	939303.44 (769873.56– 1139743.9)	1311109.6 (1115869.6– 1534459.9)	39.58

***Brazil***	1463.32 (1240.20– 1711.31)	912.36 (788.20– 1048.06)	–37.65	1918996.9 (1616752.4– 2249374.3)	2164570.3 (1868033.1– 2483588.8)	12.80

***Honduras***	1903.46 (1616.01– 2215.56)	904.78 (776.29– 1054.24)	–52.47	66869.28 (56657.88– 78305.21)	76312.81 (65038.65– 88780.77)	14.12

***Ecuador***	1965.65 (1647.37– 2313.59)	776.74 (653.95– 928.24)	–60.48	167052.69 (139128.86– 197316.98)	132898.13 (111731.95– 159078.03)	–20.45

***Guatemala***	1967.54 (1667.02– 2316.64)	712.89 (605.96– 835.04)	–63.77	121036.18 (101590.44– 143467.08)	110211.03 (92889.24– 129231.16)	–8.94

***Nicaragua***	1559.28 (1313.79– 1843.13)	668.67 (566.27– 779.08)	–57.12	44839.46 (37587.15– 53213.13)	39908.47 (33606.16– 46667.06)	–11.00


Similar to the prevalence trend, global deaths per 100,000 population due to CD decreased from (0.31; 95% UI = 0.14–0.37) in 1990 to (0.12; 95% UI = 0.07–0.21) in 2019. Furthermore, a 15.6% decrease in the absolute number of deaths was observed in this period (11,235 deaths due to CD estimated in 1990 compared to 9487 in 2019) (Supplementary Figure 3). Brazil was the country with the largest number of deaths due to CD during this period (7903 in 1990 and 6523 in 2019). On the other hand, Bolivia had the largest age-adjusted death rate per 100,000 population both in 1990 (10.93; 95% UI = 2.29–23.49) and in 2019 (7.27; 95% UI = 1.58–17.19) (Supplementary Figure 4).

The global disability-adjusted life-years (DALYs) due to CD were 360,872 (95% UI = 153,746–450,827) in 1990 and 275,377 (95% UI = 184,453–459,354) in 2019; representing an important decrease of around 23.7% DALYs over the evaluated period. Likewise, the global age-standardized DALY rates decreased from 8.51 (95% UI 3.72–10.53) in 1990 to 3.34 (95% UI 2.25–5.57) in 2019 (Supplementary Figure 5). The countries with the highest DALY rates per 100.000 population in 1990 were Bolivia (339.9; 95% UI 143.26–531.52) and Brazil (245.5; 95% UI 80.79–312.29), while in 2019 Bolivia (183.8; 95% UI 64.92–328.59) and Venezuela (92.6; 95% UI 62.26–169.48) occupied the first two places (Table 2).

**Table 2:** Top 10 countries according to the age-standardized disability-adjusted life-year rates (per 100,000 population) due to Chagas disease in 2019.


COUNTRY/STATE	DALY RATE PER 100,000 POPULATION IN 1990	DALY RATE PER 100,000 POPULATION IN 2019	PERCENTAGE CHANGE 1990–2019 (%)	DALYS (NUMBER) 1990	DALYS (NUMBER) 2019	PERCENTAGE CHANGE 1990–2019 (%)

***Bolivia (Plurinational State of)***	339.96 (143.26 – 531.52)	183.76 (64.92 – 328.59)	–45.95	12503.39 (5668.15 – 18939.51)	16882.47 (6156.26 – 29128.09)	35.02

***Venezuela (Bolivarian Republic of)***	228.02 (99.96 – 285.06)	92.55 (62.26 – 169.48)	–59.41	23314.70 (10840.66 – 28919.13)	27037.40 (18134.19 – 50564.87)	15.97

***Brazil***	245.46 (80.79 – 312.29)	72.32 (44.93 – 125.48)	–70.54	256380.58 (81679.58 – 328720.59)	174194.22 (109039.60 – 302974.38)	–32.06

***Argentina***	116.02 (69.25 – 184.24)	45.77 (28.66 – 119.24)	–60.54	36724.31 (21979.75 – 58618.17)	23552.58 (14648.12 – 61744.26)	–35.87

***Paraguay***	55.93 (25.82 – 72.47)	26.21 (15.76 – 44.77)	–53.14	1358.92 (659.02 – 1748.67)	1541.27 (947.25 – 2653.88)	13.42

***Chile***	40.65 (29.20 – 53.75)	18.01 (12.87 – 24.02)	–55.71	4594.70 (3259.73 – 6165.39)	4195.56 (2987.86 – 5587.42)	–8.69

***Honduras***	24.53 (14.65 – 33.85)	15.80 (7.81 – 24.79)	–35.61	607.24 (384.35 – 843.46)	1003.25 (538.33 – 1518.92)	65.21

***El Salvador***	26.84 (17.48 – 39.51)	12.67 (8.19 – 21.96)	–52.79	918.93 (594.81 – 1293.11)	759.50 (489.69 – 1314.18)	–17.35

***Colombia***	12.26 (9.12 – 21.27)	10.34 (4.79 – 15.53)	–15.67	2617.90 (1966.51 – 4186.43)	5426.21 (2529.02 – 8181.17)	107.27

***Mexico***	12.64 (8.35 – 17.93)	10.22 (6.79 – 14.28)	–19.11	6933.23 (4479.72 – 9991.35)	12533.67 (8303.68 – 17462.50)	80.78


### Burden by regions

**Figure 2:**
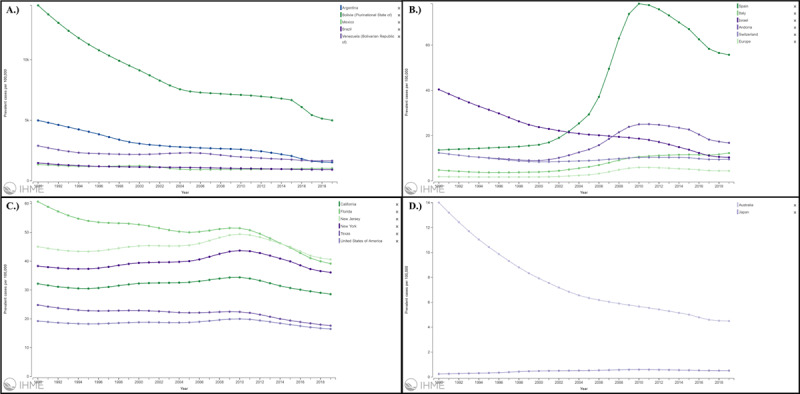
Chagas Disease age-standardized prevalence rate by year in the **A.)** top 5 Latin American countries, **B.)** top 5 European region countries, **C.)** top 5 U.S. states, and **D.)** Australia and Japan from 1990 to 2019. Source: https://vizhub.healthdata.org/gbd-compare/.

As expected, the Latin American region has suffered the highest burden of the disease, with a total of 6,354,220 individuals with CD diagnosis by 2019, accounting for a prevalence rate per 100,000 population of 933.76 (95% UI 817.66–1064.29). Nevertheless, this prevalence rate has dropped in the last 30 years, as shown in Supplementary Figure 6. A different scenario occurred in North America’s region, which showed a progressive increase in the prevalence rate until 2010, followed by a sustained drop (Supplementary Figure 7). Finally, CD prevalence in the European region showed a similar trend, with a substantial increase from 1990 until 2010, followed again by a drop in the cases rate after this point (Supplementary Figure 8). Figure 2 shows a summary of the prevalence trends in the most affected countries/U.S. states by region. Table 1 and Supplementary Table 1 summarize the prevalence rates and numbers and their change between 1990 and 2019 by country and U.S. state.

On the other hand, DALY rates per 100,000 population showed a similar trend in the Latin American region during this period, with a sustained drop in its value from 116.72 (95% UI 48.85–145.08) in 1990 to 40.54 (95% UI 27.04–67.75) in 2019. Regarding the North American region, a steady increase in the DALY rate was observed until 2015 (0.39 DALYs per 100,000 population; 95% UI 0.28–0.5), followed by a steep decline thereafter. Finally, a relatively stable trend was observed in the European region, with an initial drop in the DALY rate after 1994 followed by a period without significant changes in this value.

**Figure 3:**
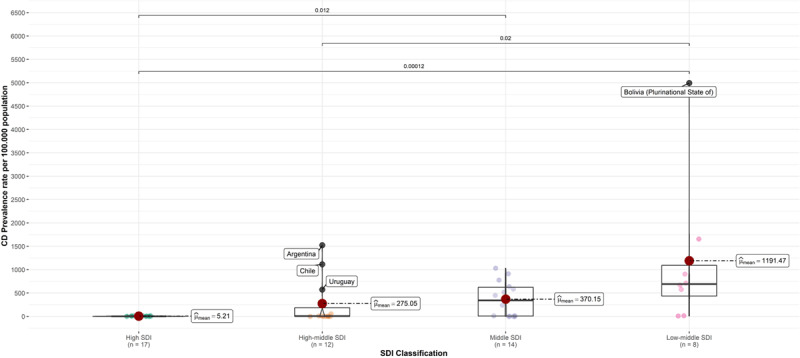
Chagas Disease (CD) prevalence rate per 100,000 population in 2019 according to the sociodemographic index (SDI) classification. Source: https://vizhub.healthdata.org/gbd-compare/.

Finally, we evaluated a potential association between the sociodemographic index (SDI) classification of the assessed countries and the CD prevalence rate. Countries classified as low-middle SDI had the highest CD prevalence rate (median value: 691 cases per 100,000 population), followed by those in the middle SDI group (median value: 345 cases per 100,000 population). On the other hand, the countries in the high-middle and high SDI groups had significantly lower prevalence rates (7.88 cases and 2.15 cases per 100,000 population, respectively). Figure 3 summarizes the CD prevalence rate of the evaluated countries according to the respective SDI category, highlighting a significantly larger rate in the low-middle SDI group compared to the high-middle (p-value: 0.02) and high SDI (p-value < 0.001) groups, along with a significantly higher prevalence in the middle SDI group compared to the high SDI one (p-value: 0.012). Finally, Figure 3 also highlights potential outliers among the classification groups, including Argentina, Chile, and Uruguay in the high-middle SDI group and Bolivia in the Low-middle SDI group.

### Burden by age and sex

Supplementary Figure 9 summarizes the estimated prevalence rate per 100,000 population by age and sex in 1990 and 2019. At first, a male-to-female ratio of around 0.9:1 was observed by 1990, having women a slightly higher prevalence mainly across ages below 60 years with a peak in the age group of 45 to 49 years (220.48 per 100,000 population [95% UI: 192.32–251.63]) and a progressive reduction in the prevalence rate after this point. On the other hand, males showed a peak in the age group of 95 plus years (302.31 per 100,000 population [95% UI: 259.04–350.80]), a trend that remained until 2019. Nevertheless, the global male-to-female ratio varied during this period, resulting in a value of around 1.1:1 by 2019.

**Figure 4:**
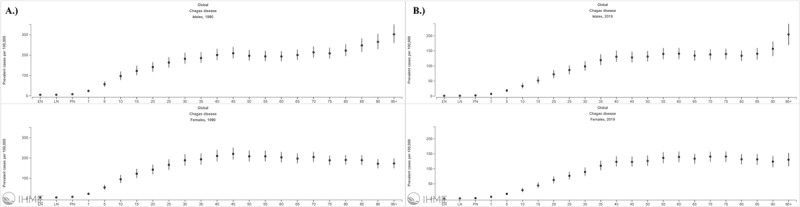
Sex- and age-specific rates per 100,000 of disability-adjusted life-years (DALYs) due to Chagas Disease (CD) worldwide in 2019. Source: https://vizhub.healthdata.org/gbd-compare/.

Furthermore, by 2019, the highest DALY rates were observed among individuals above 84 years, highlighting a peak in the age group of 95 plus years (25.65 DALYs/100,000 population [95% UI: 15.04–47.93]) as observed in Figure 4. By the same year, the estimated age-standardized DALY rate in males almost doubled the one observed in females (4.17 DALYs/100,000 population [95% UI 2.36–7.25] vs. 2.59 DALYs/100,000 population [95% UI 1.49–4.88], respectively), revealing a male-to-female ratio of around 1.6:1. Figure 4 shows a similar trend of DALYs by sex across age groups, highlighting a higher burden in men in almost all categories and a male-to-female ratio that increases progressively as age increases.

It is relevant to highlight the differences between regions regarding the gender gaps observed. For example, we observed a large DALYs rate male-to-female ratio in Latin America, which remained nearly unchanged during the study period (1.68:1 in 1990 and 1.69:1 in 2019), evidencing that men have substantially higher DALY rates than women in this region (Supplementary Figure 10). On the other hand, gender differences in the North America’s region were less stable, with a DALY rate male-to-female ratio of 1.22:1 in 1990 that was followed by a peak by 2015 (2.43:1) and a subsequent reduction until 2019 (1.5:1) (Supplementary Figure 11). In parallel with the observed reduction in the rate of DALYs in the European region, a reduction in the gender gap was observed in this region, from 1.64:1 in 1990 to 1.31:1 in 2019 (Supplementary Figure 12).

Finally, a sustained decrease in the rate of deaths attributed to CD over time was observed in both sexes. Nevertheless, males had a higher mortality rate per 100,000 population across the almost three decades of analyzed estimates, with a male-to-female ratio of 1.6:1 in 1990 and 1.5:1 in 2019 (Supplementary Figure 13). Moreover, the countries with the largest male-to-female ratio for mortality in 2019 were El Salvador (2.4:1), Ecuador (2.4:1), and Argentina (2.2:1).

## Discussion

According to the GBD data, there has been a sustained decrease in Chagas Disease burden, measured in prevalence, deaths, and DALYs due to CD worldwide from 1990 to 2019. Nevertheless, this trend has not been the same for every affected region, with a more abrupt reduction of the burden in Latin America, while data from North America and Europe reveal a more stable trend, with increasing prevalences until 2010. Moreover, we highlighted significant differences in the burden by sex, being men the most affected, age, with the elderly having the highest burden of the disease, and sociodemographic index (SDI), with countries with the lowest SDI values having the highest prevalence of the disease. These findings have relevant public health implications; however, they need to be assessed in light of the current published evidence estimating CD’s burden in each region.

The main finding of the present study is the substantial reduction in the prevalence of the disease and the burden attributed to it worldwide. Although interregional differences were observed, the reduction in the number of cases and deaths secondary to CD may reflect the impact of decades of public policies aimed at its mitigation. Initially, the elimination of vector-borne transmission became the goal of governments of endemic countries, which led vector eradication initiatives since the 1970s. These programs included the restoration of homes to prevent triatomine infestation, as well as the use of synthetic insecticides for vector control, among others [11]. The impact of these programs was initially measured in the number of municipalities with triatomine infestation, and in the case of Brazil, in which a significant decrease in this number was observed, especially in those infested by *Triatoma infestans*. This decrease was also associated with a reduction in the number of cases of acute Chagas disease and in the prevalence of infection in children under five years of age [12]. Furthermore, climate change has been postulated as one of the potential determinants of CD transmission in the region and the world. Despite this, studies that have analyzed the impact of temperature change on parameters such as the life cycle, fertility and fecundity of vectors have yielded divergent results [13,14,15,16,17]. Therefore, there is a clear need for studies that comprehensively evaluate the relevant ecological and climatic factors in the parasite-vector interaction.

The GBD Study estimations summarized in the present study differ vastly from what has been calculated by other studies published in the literature. At first, the prevalence reported in the U.S. (in 1990 and 2019) was substantially lower than that estimated by the study of Bern et al., which estimated a prevalence number of 300,167 cases of individuals with *T. cruzi* infection living in the U.S. by 2009, with 30,000–45,000 cases of cardiomyopathy [6]. Similar results were reported in the study of Manne-Goehler et al., with a national estimate of 238,091 cases as of 2012 [9]. The differences in these estimations could be related to the varied underlying estimates of immigration and seroprevalence by region used in each study. For example, the study of Bern et al. estimated the number of Latin American migrants from CD endemic countries using information from the Pew Hispanic Center and the reports of unauthorized immigrant populations published by the Department of Homeland Security [6]. On the other hand, Manne-Goehler et al. used information from the American Community Survey to estimate the number of residents born in CD endemic countries [9]. Both studies relied on data from the World Health Organization to estimate the prevalence of *T. cruzi* infection in 2006 and 2015 [6,9].

The results of this study also differ from previous estimations of CD prevalence in the European Region. For example, the study of Gascon et al. estimated that a total of 47,743 foreign-born individuals infected by *T. cruzi* could be living in Spain by 2009 [18]. This study also used data from the PAHO/WHO to estimate CD’s prevalence and burden in each endemic country. On the other hand, the systematic review of Navarro et al. estimated that there were around 34,202 Bolivian immigrants with *T. cruzi* infection living in Spain by 2010, highlighting a much higher total prevalence when considering immigrants from other nationalities [19]. Nevertheless, this review included studies conducted in hospital-based settings; therefore, the results can potentially overestimate the actual population prevalence due to selection bias. A way to prevent this overestimation is performing separate estimates from antenatal care screenings, primary health care/community-based studies, and even blood bank data. However, it is important to highlight that studies based on blood bank data tend to underestimate CD prevalence [20].

Considering that the PAHO/WHO estimates may not be the only sources of information regarding the prevalence of CD in endemic regions, we reviewed the studies that have provided national estimates of this prevalence in endemic countries, evaluating three studies assessing the nationwide prevalence of CD in endemic countries. Our results suggest that even the previously mentioned studies, which relied on PAHO/WHO prevalence estimates, could underestimate the actual prevalence of CD in non-endemic countries, as the estimates from the studies included have suggested higher prevalence values compared to those reported by the PAHO/WHO in 2006 and 2015 [21,22]. At first, the meta-analysis of Arnal et al. estimated the seroprevalence of *T. cruzi* infection in Mexico at 3.38% (95%CI 2.59–4.16), a value almost 5-fold higher from the one reported by the PAHO/WHO in 2015 (0.77%) and tripling the value of the 2006 estimations (1.03%) [23]. Moreover, the meta-analysis of Martins-Melo et al., published in 2014, estimated a prevalence of CD of 2.4% (95% CI: 1.5–3.8) in Brazil by using studies published after 2000. This prevalence doubles the estimates reported by the PAHO/WHO in 2006 (1.02%) and triples the ones of the 2015 report (0.61%) [24]. A higher prevalence than the PAHO/WHO statements was also reported by Olivera MJ et al. in Colombia [25]. Considering only the estimated migrants from these three countries (Colombia, Brazil, and Mexico) in the U.S. (according to the 2019 American Community Survey) and the updated prevalence data, an estimate of 406,748 individuals from these endemic countries that are currently living in the U.S. could be infected with the parasite. Adding this value to the estimated number of CD cases in immigrants from other endemic countries using the PAHO/WHO data on CD prevalence would yield an estimate of 472,397 CD cases in the U.S. alone. This updated data suggest that the burden of the disease could be even higher than what has been estimated before, highlighting the importance of having new up-to-date national estimates on CD prevalence in endemic regions.

Furthermore, important differences in the burden by sex were observed, highlighting a higher burden of CD in males. At first, prevalence differences can be explained by biological, sociocultural, and behavioral factors, such as the exposure to the vector as a result of occupational activities, as there is evidence that suggests a potentially relevant role of vector transmission in working areas [26,27]. Moreover, male sex has been identified as an independent risk factor for progression from the indeterminate form of the disease to chronic Chagas cardiomyopathy, which may promote the diagnosis due to the onset of HF symptoms [28]. Regarding the burden of the disease, several studies have observed a higher risk of morbimortality in males due to cardiac complications of CD [29]. Recently, significant differences in myocardial damage by sex have been observed, suggesting potentially different pathophysiological mechanisms involved in the development and progression of cardiomyopathy. In the study of Assunção Jr et al., in which gender differences in myocardial damage assessed by cardiovascular magnetic resonance were evaluated, a significant association between gender and myocardial dysfunction was observed, with men showing greater myocardial fibrosis and lower LV ejection fraction values. Moreover, the transmural pattern of involvement of the myocardial tissue along with the myocardial gray zone were more prevalent among males [30]. In addition, access to health services may represent a relevant factor differentiating outcomes by sex, highlighting the hesitancy of males in searching for health services until the appearance of severe incapacitating symptoms or complications, such as chronic Chagas cardiomyopathy [31,32,33]. This leads to a late diagnosis of the disease, potentially explaining the higher prevalence in older ages in males. On the other hand, the higher DALY rate in the elderly may be related to the chronic nature of the disease and the increasing prevalence of chronic comorbidities in older ages [34].

Finally, an aspect that has not been thoroughly assessed is the DALYs associated with the disease. The studies of Martins-Melo et al. used the GBD study data to estimate the burden of CD and other neglected tropical diseases in Brazil, highlighting an important reduction in the burden from 1990 to 2016, as reported in our study [35,36]. On the other hand, the burden associated with CD in terms of DALYs has not been studied in non-endemic regions, with the only information available coming from the computational simulation model of Lee et al., which estimated that each affected individual incurs in US$474 in health-care costs and 0·51 DALYs annually, reflecting an estimated global annual burden of $627.46 million in health-care costs and 806,170 DALYs. Regarding the burden by region, this study estimated a net present value of DALYs in the U.S. and Canada of 1,123,552 (range 83,643–4,470,747), which is similar to what we reported using the GBD data [10].

### Limitations

The present study suffers from several limitations. At first, it was based on the GBD study data to generate the estimates provided. Although the methodology of the GBD is considered reliable, we must acknowledge that it can be limited by the quality of the available data, which can vary across countries and time, relying on mathematical models to assess the burden in countries with limited information. In this context, the GBD used the data of CD prevalence by country from the Pan American Health Organization/World Health Organization to estimate the number of *T. cruzi* infection cases; however, these reports may provide inaccurate estimations, as some were based on extrapolations from mathematical models using vector distribution or from blood donor screening programs. Specifically, the high prevalence of asymptomatic individuals with CD, even in those with cardiomyopathy, may directly impact the prevalence estimates.

Moreover, the information reported was based on multiple assumptions, for example, that the state-wise distribution of foreign-born persons is similar for all countries within a region and that the prevalence of CD in the immigrant population is similar to that of the country of origin. Furthermore, the global trends observed in this study may not correlate with micro-level trends for some regions, such as the United States of America, with may show an important subnational variability in the burden of CD. Therefore, the CD trends observed in this study must be interpreted with caution, especially for those estimates with larger uncertainty intervals.

## Conclusions

The global burden of CD has been dramatically changing in the last decades, with a sustained decrease in the number of cases and the disease’s burden. Despite the vast majority of the cases still concentrate in Latin America, a significant prevalence is reported in developed countries of North America and the European Region. Although it provides valuable detailed information for health policymakers, data from the GBD study can potentially underestimate the prevalence of the disease in non-endemic countries. There is a relevant need to carry out representative seroprevalence studies and screen migrant populations to provide more precise estimates of the disease prevalence in non-endemic countries. Moreover, raising the awareness of this neglected tropical disease in these regions is critical for mitigating its impact and allowing earlier diagnoses, therefore, potentially improving the clinical outcomes of CD-infected patients.

## Data Accessibility Statement

To download the data used in these analyses, please visit the Global Health Data Exchange GBD 2019 website (http://ghdx.healthdata.org/gbd-results-tool).

## Additional File

The additional file for this article can be found as follows:

10.5334/gh.1150.s1 Supplementary File. Supplementary figures 1 to 13 and Tables 1 and 2.
