# Supplementary material for: Global, Regional, and National Trends of Chagas Disease from 1990 to 2019: Comprehensive Analysis of the Global Burden of Disease Study
Source: Glob Heart. 2022 Aug 24;17(1):59. doi: 10.5334/gh.1150 (PMC9414802; doi:10.5334/gh.1150)

Global, Regional, and National Trends of Chagas Disease from 1990 to 2019: Comprehensive Analysis of the Global Burden of Disease Study

Supplementary File

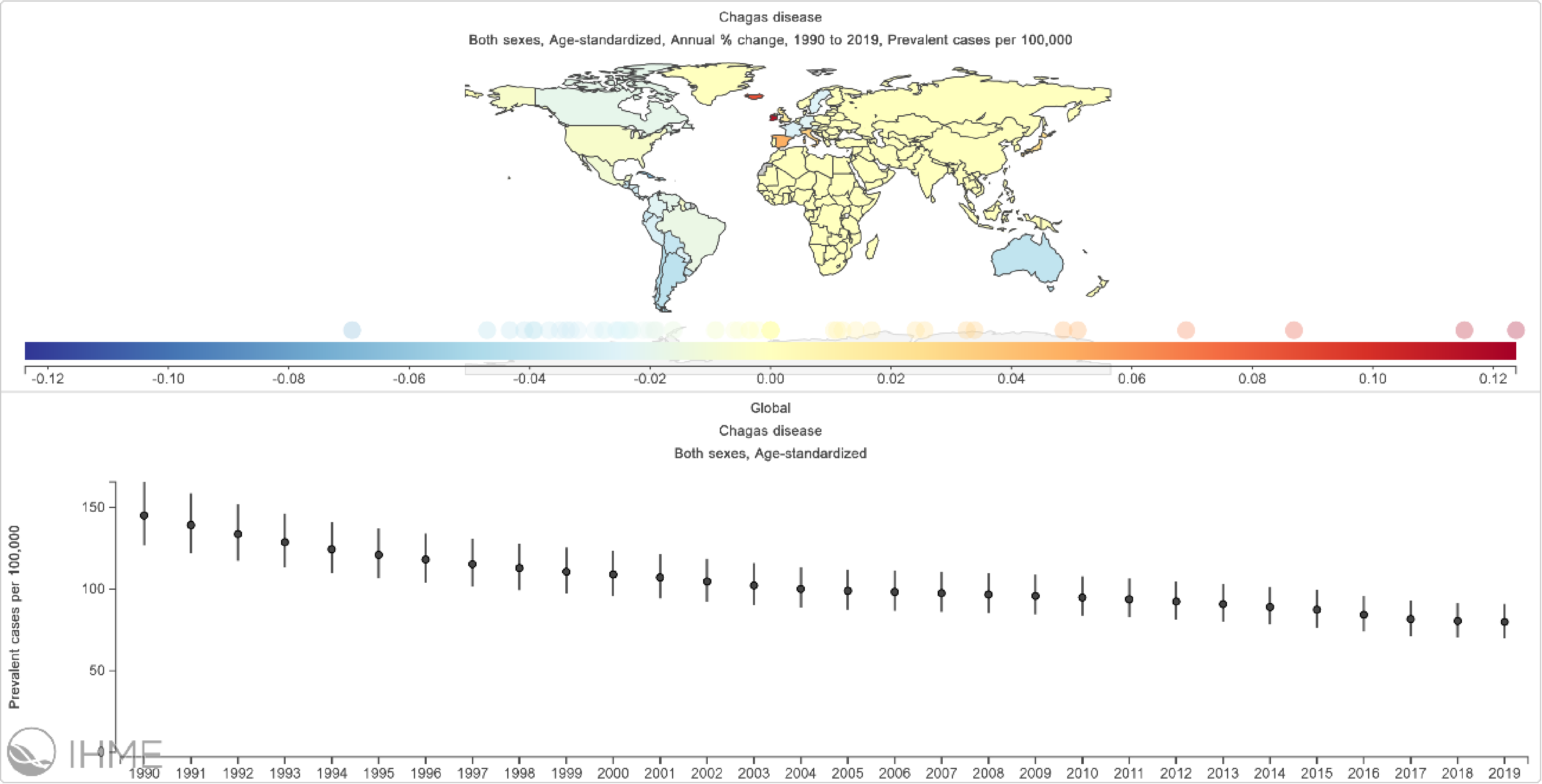

Supplementary Figure 1. Global Chagas Disease (CD) age-standardized prevalence rate trends from 1990 to 2019. **Top:** Annual % change in the age-standardized prevalence rate per country. **Bottom:** Mean estimates and uncertainty intervals of the global age-standardized prevalence rate per year.

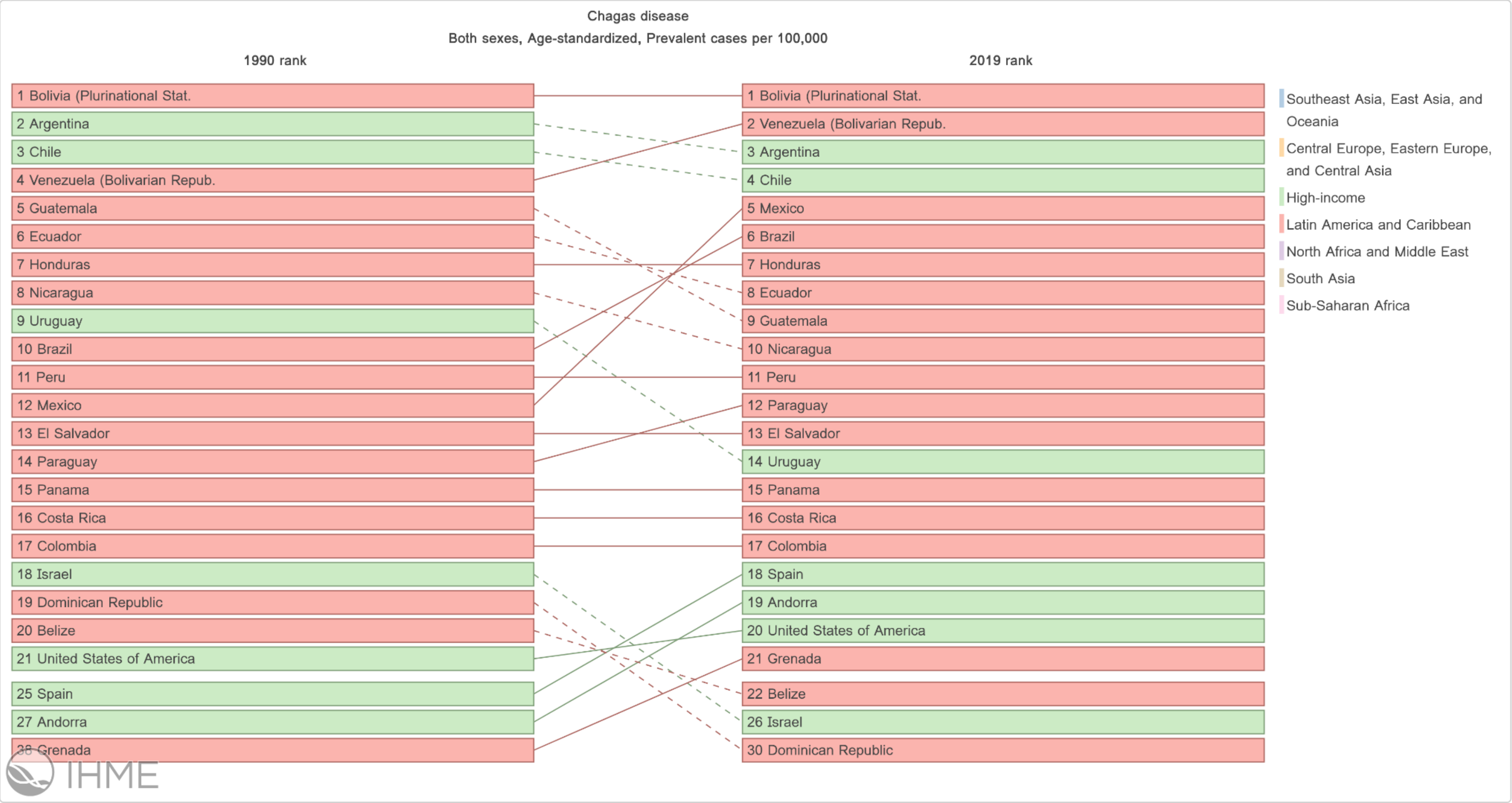

Supplementary Figure 2. Ranking of countries by Chagas Disease prevalence rate per 100,000 population in 1990 and 2019.

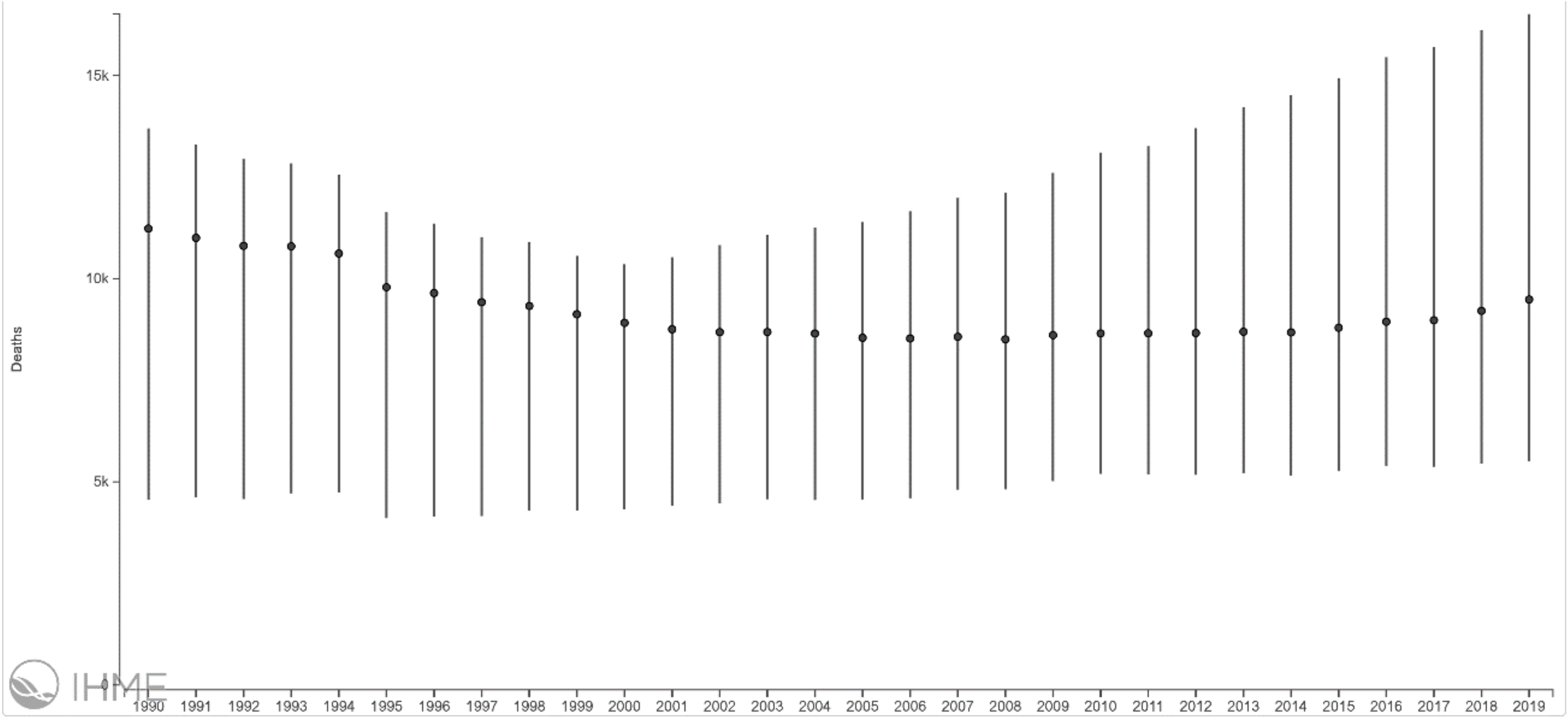

Supplementary Figure 3. Global Chagas Disease (CD) absolute number of attributed deaths from 1990 to 2019.

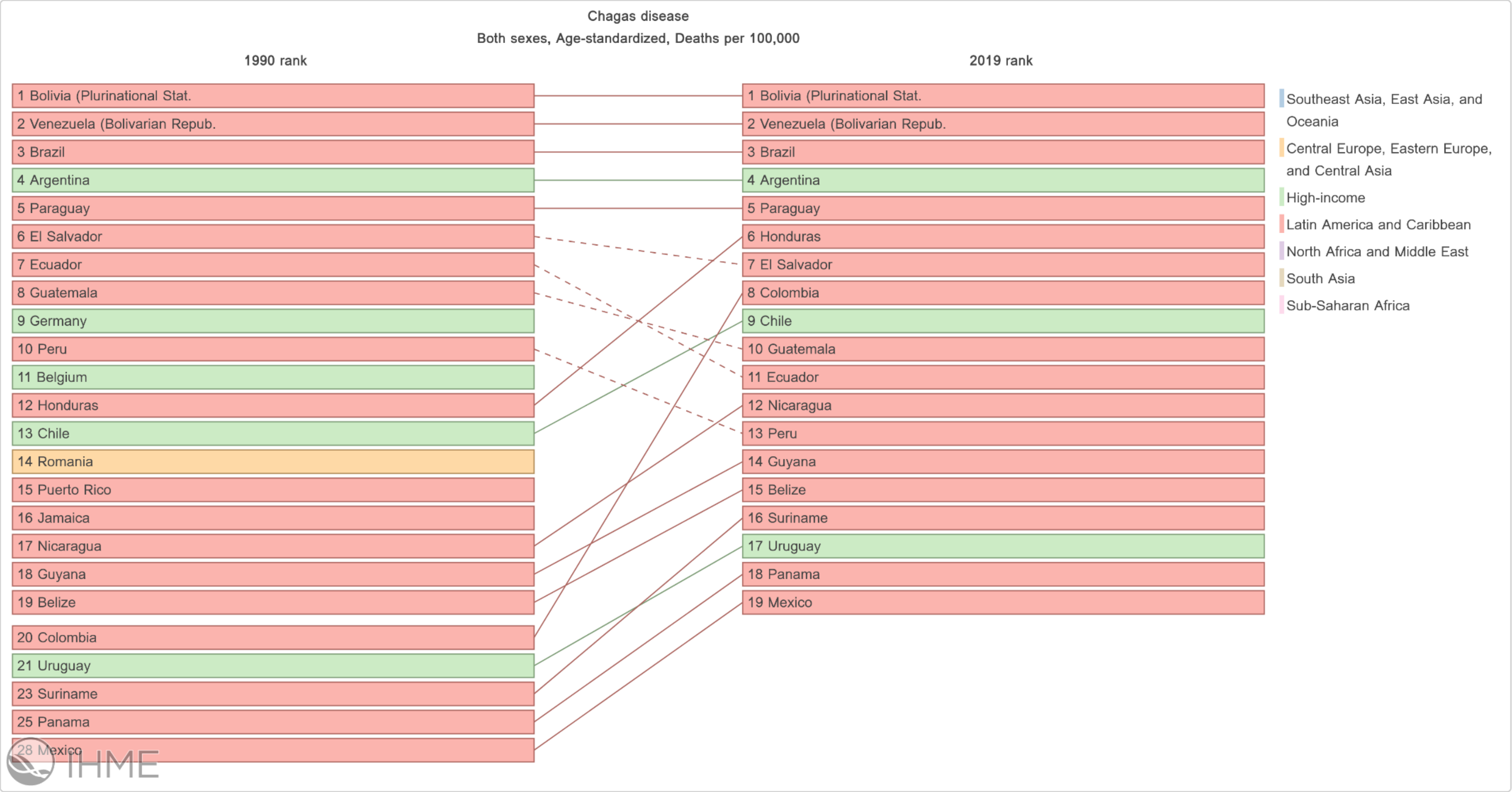

Supplementary Figure 4. Ranking of countries by Chagas Disease death rate per 100,000 population in 1990 and 2019.

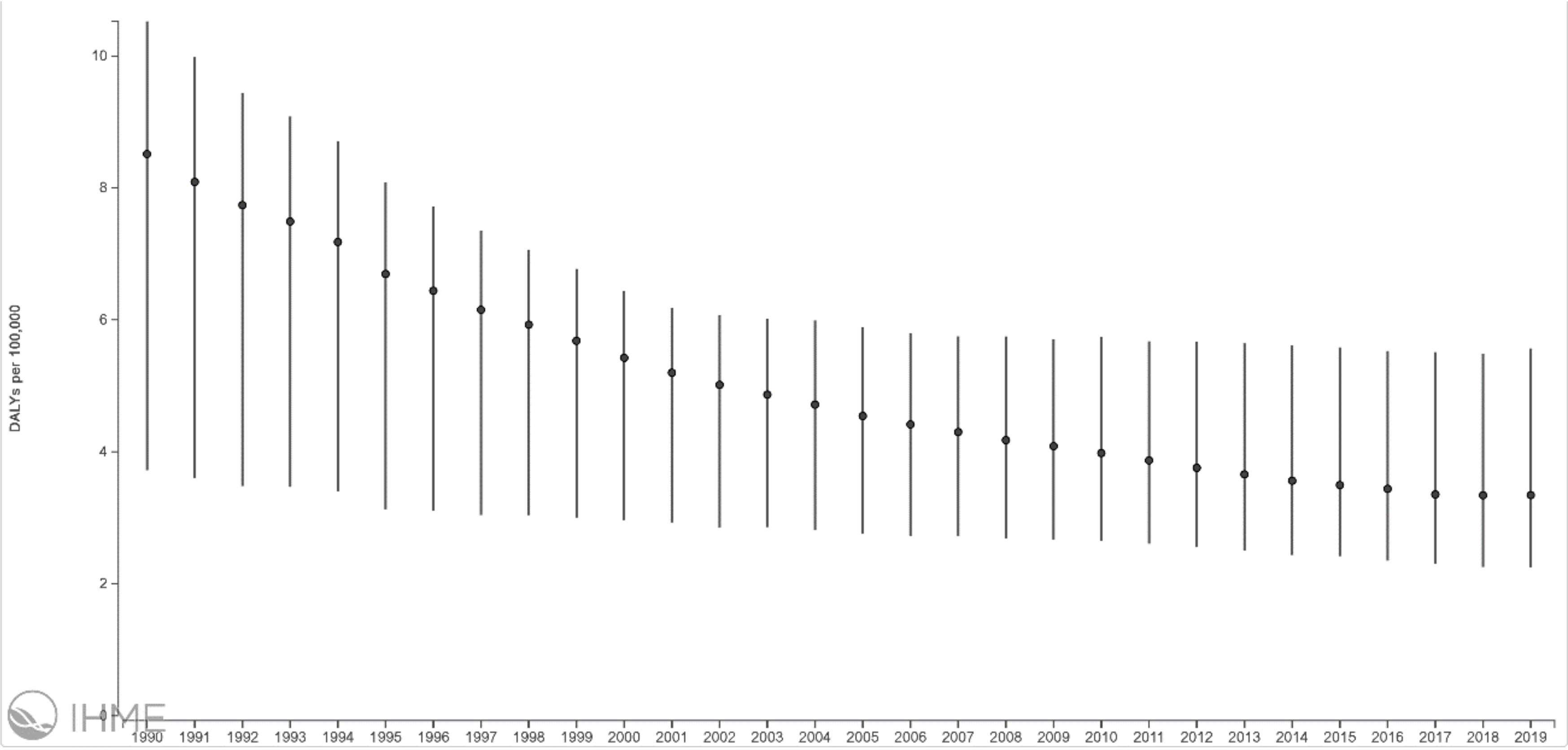

Supplementary Figure 5. Global Chagas Disease (CD) disability-adjusted life-years (DALYs) age-standardized rate from 1990 to 2019.

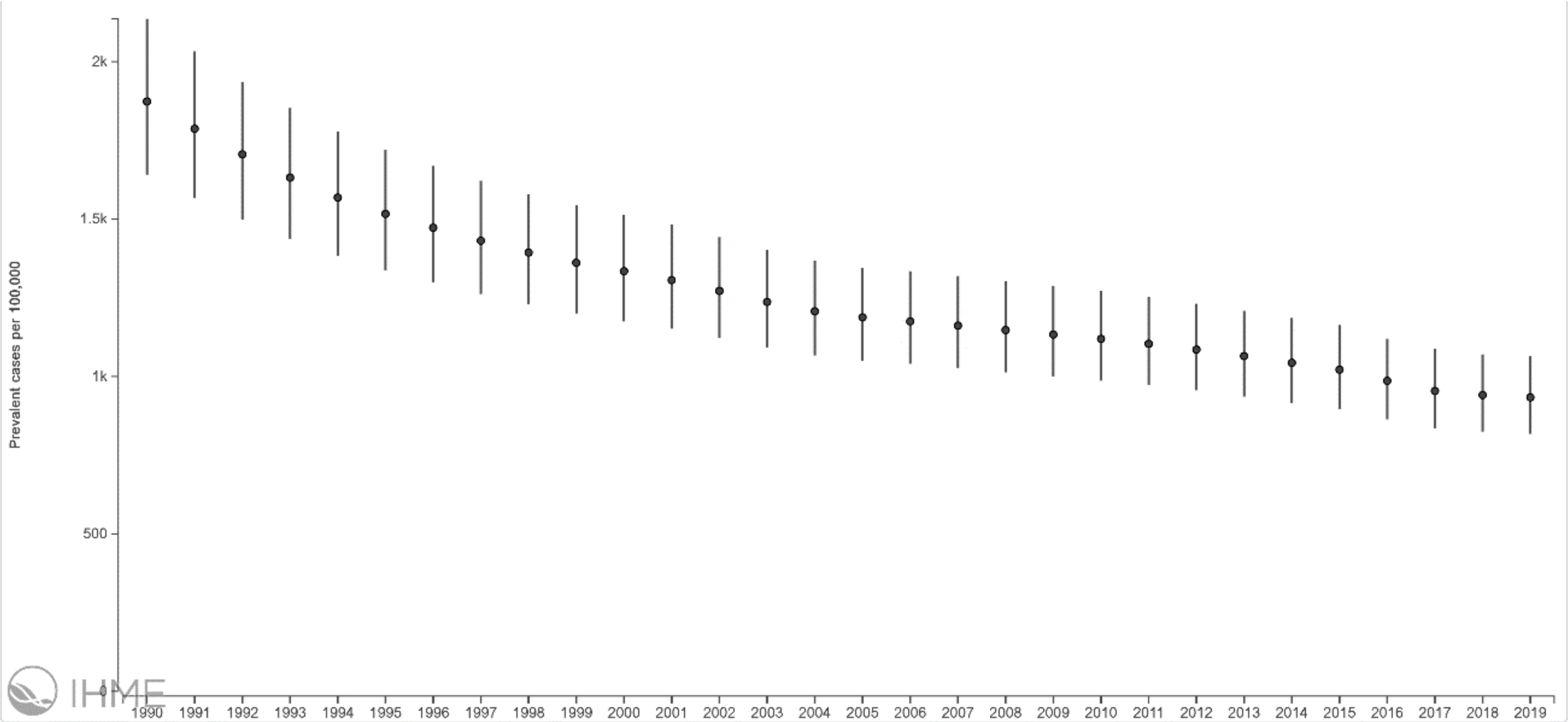

Supplementary Figure 6. Chagas Disease (CD) age-adjusted prevalence rate per 100,000 population in the Latin American Region from 1990 to 2019.

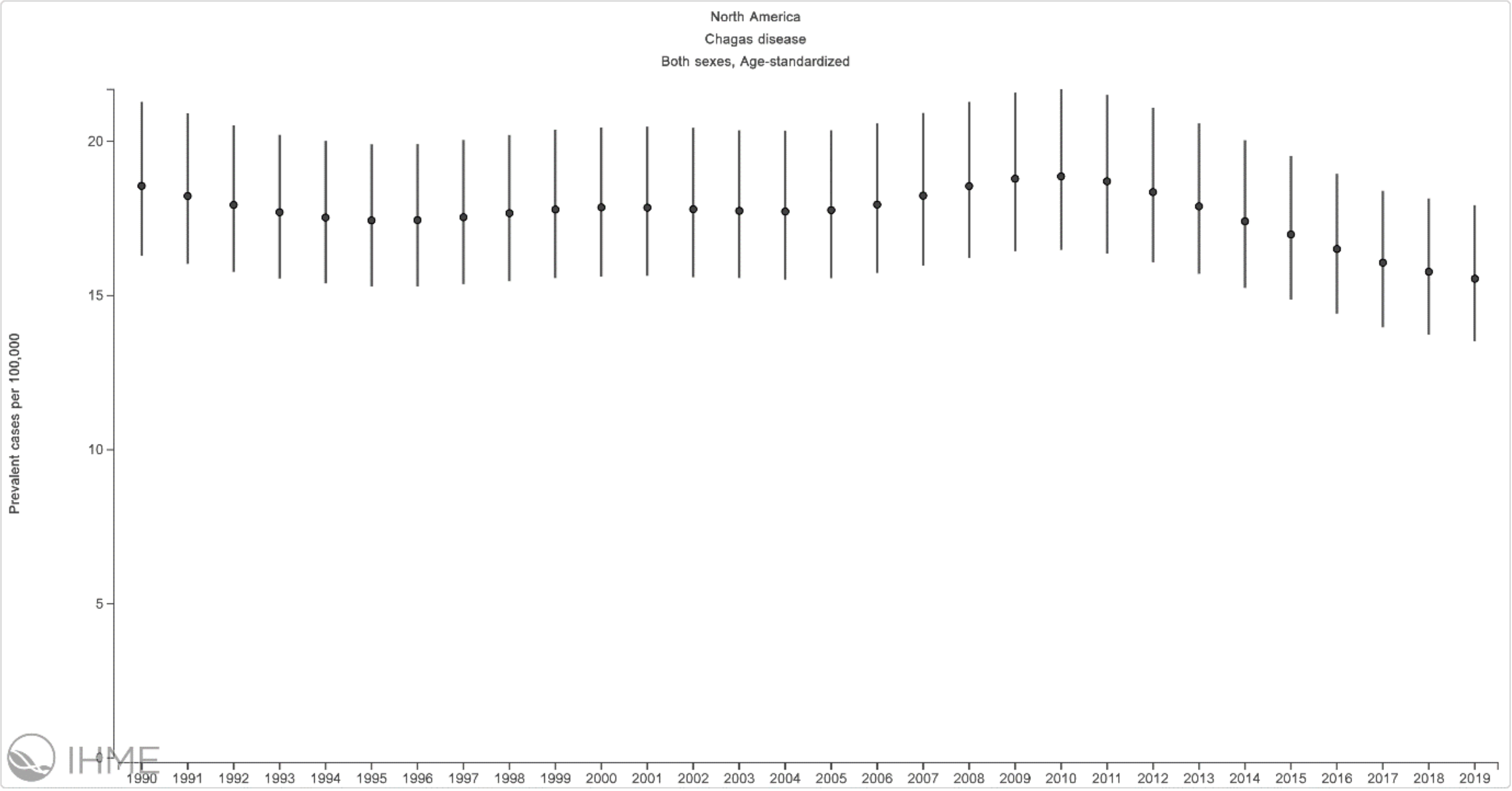

Supplementary Figure 7. Chagas Disease (CD) age-adjusted prevalence rate per 100,000 population in North America from 1990 to 2019.

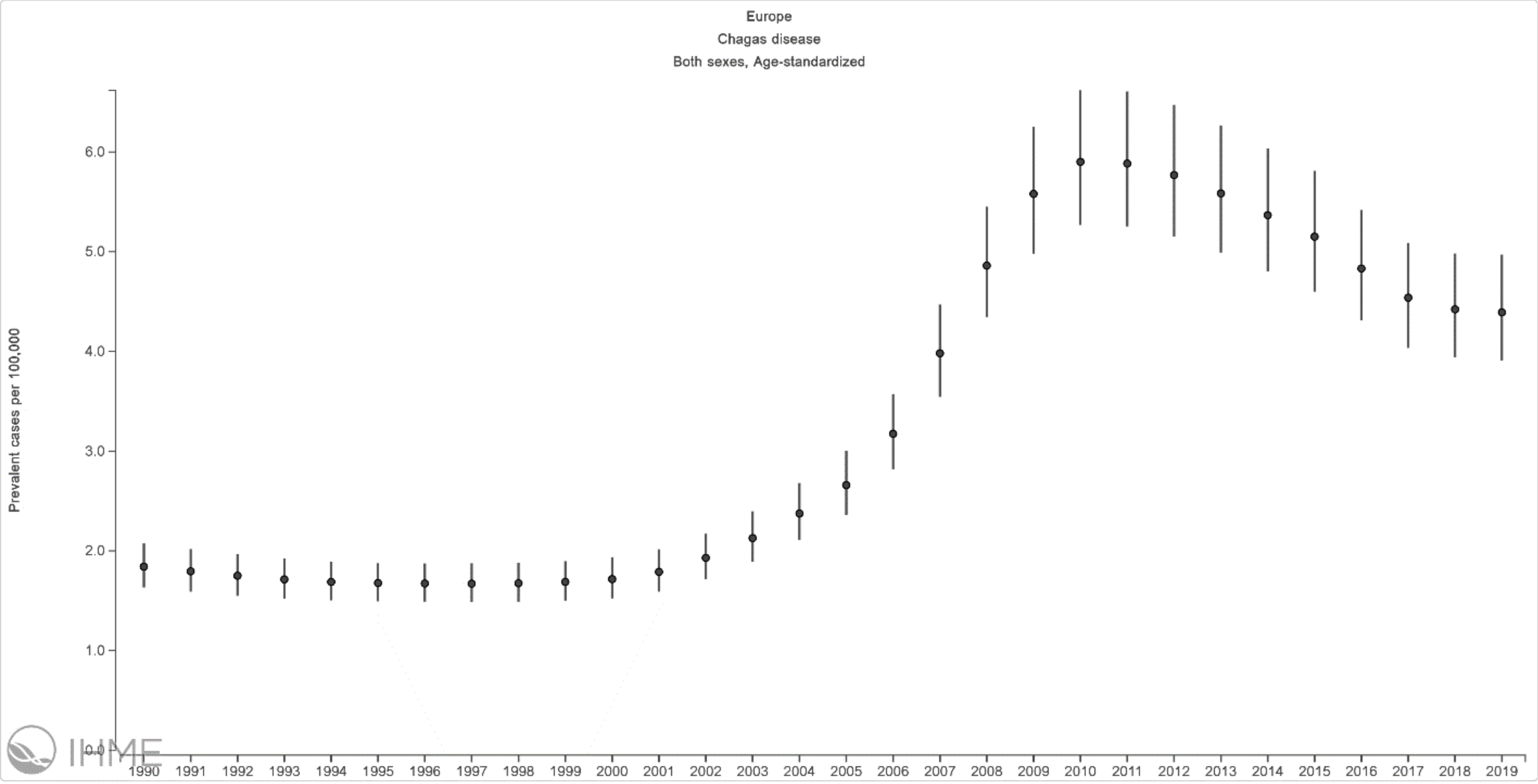

Supplementary Figure 8. Chagas Disease (CD) age-adjusted prevalence rate per 100,000 population in the European Region from 1990 to 2019.

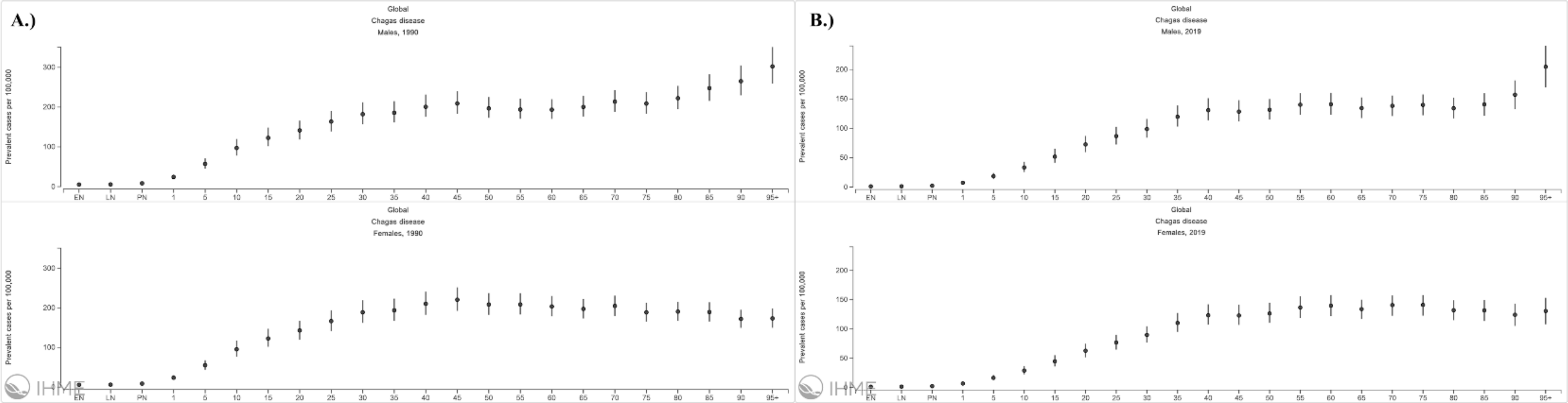

Supplementary Figure 9. Global Chagas Disease prevalence rate per 100,000 population by age and sex in A.) 1990 and B.) 2019.

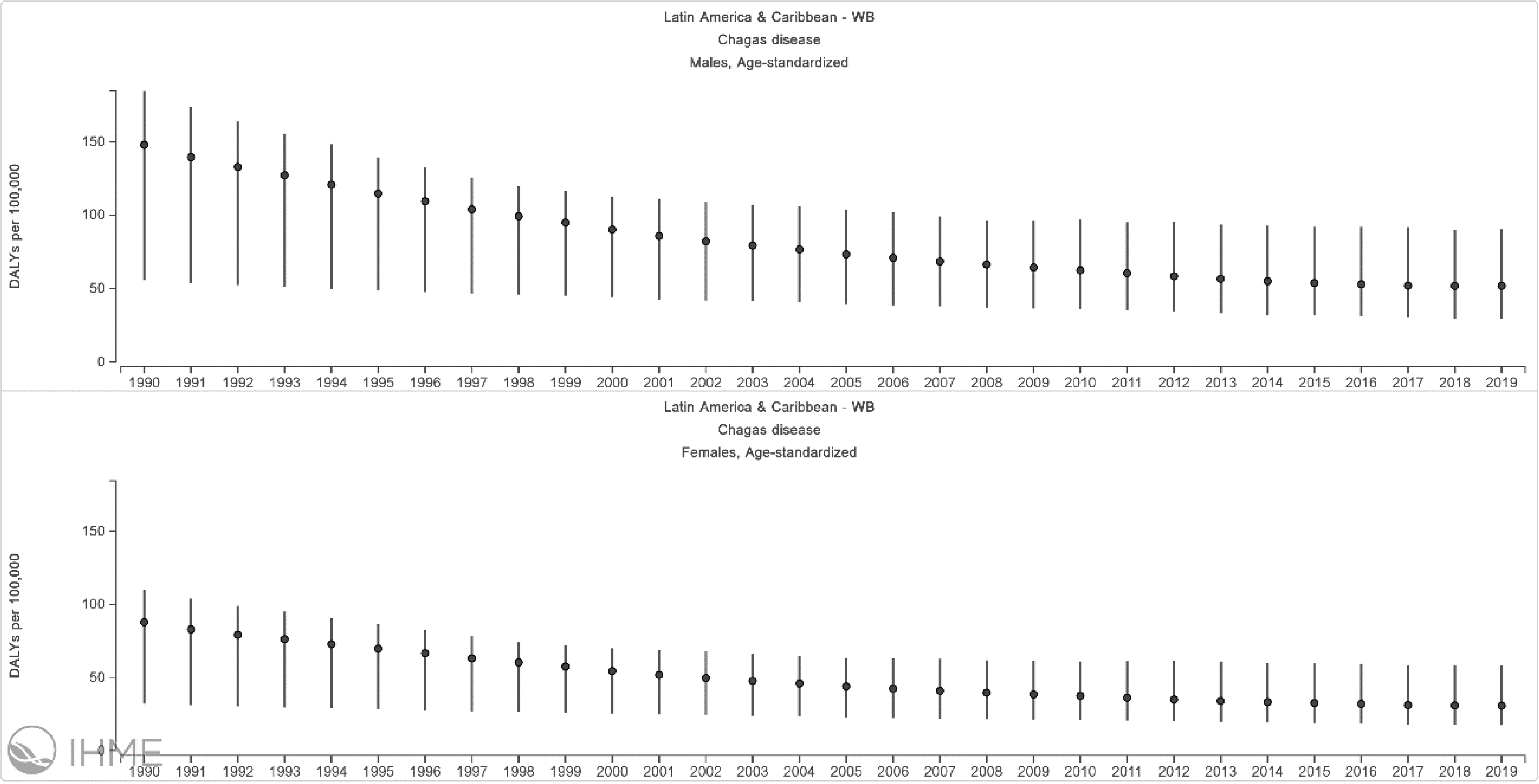

Supplementary Figure 10. Chagas Disease disability-adjusted life-years (DALYs) rate per 100,000 population in the Latin American Region by sex from 1990 to 2019.

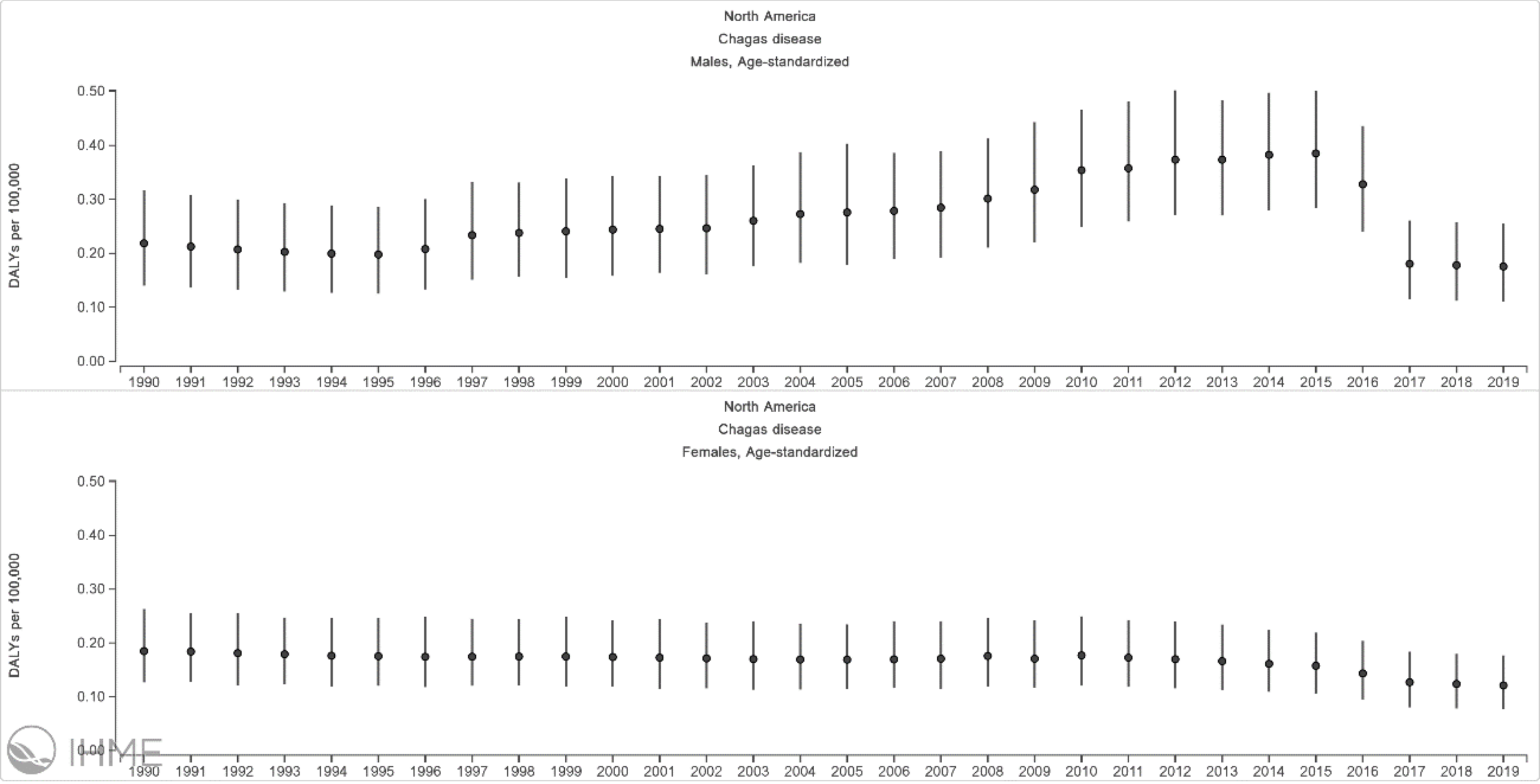

Supplementary Figure 11. Chagas Disease disability-adjusted life-years (DALYs) rate per 100,000 population in North America by sex from 1990 to 2019.

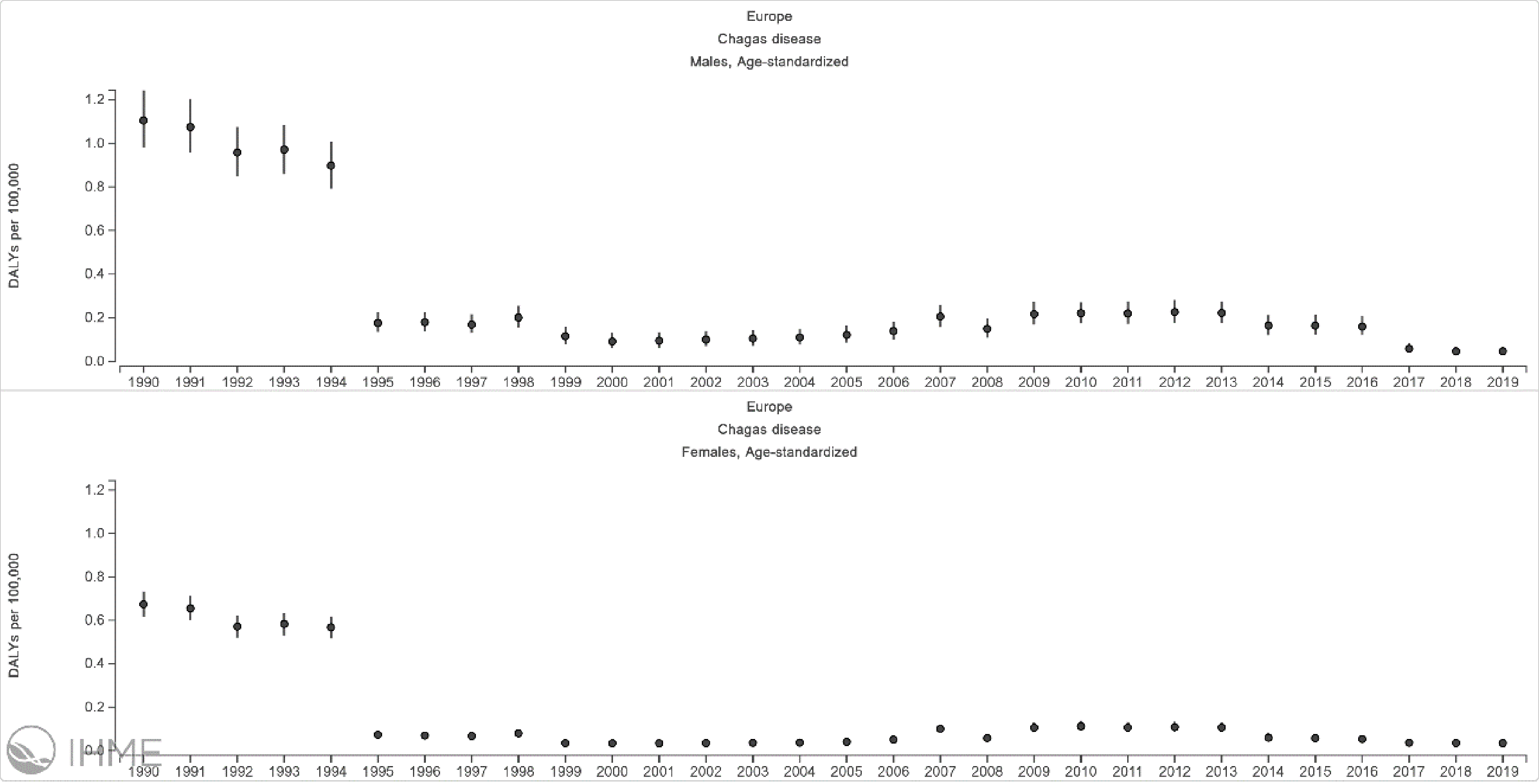

Supplementary Figure 12. Chagas Disease disability-adjusted life-years (DALYs) rate per 100,000 population in the European Region by sex from 1990 to 2019.

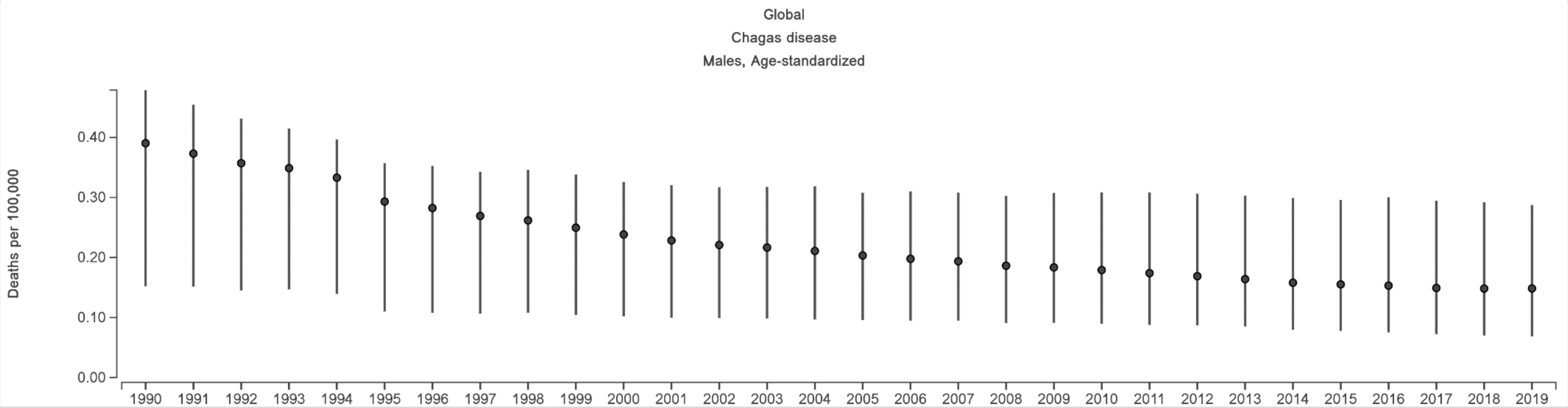
Global
Chagas disease
Males, Age-standardized
Deaths per 100,000
0.00
0.10
0.20
0.30
0.40
1990 1991 1992 1993 1994 1995 1996 1997 1998 1999 2000 2001 2002 2003 2004 2005 2006 2007 2008 2009 2010 2011 2012 2013 2014 2015 2016 2017 2018 2019

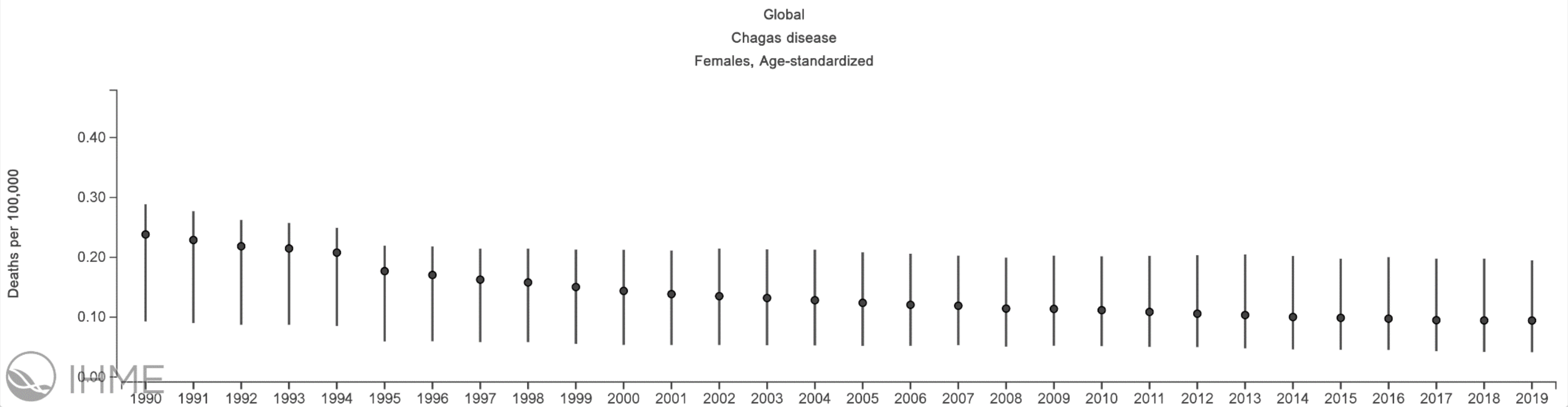
Global
Chagas disease
Females, Age-standardized
Deaths per 100,000
0.00
0.10
0.20
0.30
0.40
1990 1991 1992 1993 1994 1995 1996 1997 1998 1999 2000 2001 2002 2003 2004 2005 2006 2007 2008 2009 2010 2011 2012 2013 2014 2015 2016 2017 2018 2019

Supplementary Figure 13. Global Chagas Disease death rate per 100,000 population by sex from 1990 to 2019.

Supplementary Table 1. Number and age-standardized rate for Chagas Disease prevalence in 1990 and 2019 and the percentage change by country/US state.

Country/State	Prevalence (rate per 100,000 population) 1990	Prevalence (rate per 100,000 population) 2019	Percentage Change 1990-2019 (%)	Prevalence number 1990	Prevalence number 2019	Percentage Change 1990-2019 (%)
Andorra	12.38 (9.47- 15.75)	16.79 (11.86- 23.43)	35.62	7.85 (5.98 - 9.97)	17.48 (12.15- 24.32)	122.56
Antigua and Barbuda	0.67 (0.53- 0.86)	1.79 (0.93- 3.22)	167.16	0.35 (0.27 - 0.44)	1.85 (0.94- 3.40)	432.12
Argentina	4987.04 (4423.46- 5566.96)	1524.07 (1343.16- 1736.00)	-69.44	1622896.4 (1441816.8 - 1811864.5)	735490.88 (648796.81- 838063)	-54.68
Australia	14.01 (12.42- 15.78)	4.49 (3.92- 5.20)	-67.95	2561.94 (2270.29- 2886.15)	1302.89 (1133.42- 1496.13)	-49.14
Austria	2.21 (1.94- 2.52)	1.23 (1.04- 1.45)	-44.34	196.15 (172.17- 223.49)	132.49 (111.20- 156.01)	-32.46
Bahamas	0.33 (0.16- 0.53)	1.46 (0.96- 2.15)	342.42	0.69 (0.35- 1.06)	6.26 (4.12- 9.18)	812.67
Barbados	0.18 (0.14- 0.24)	0.17 (0.13- 0.23)	-5.56	0.47 (0.37- 0.59)	0.68 (0.52- 0.89)	46.01
Belize	22.01 (16.99- 27.96)	13.82 (10.41- 18.05)	-37.21	28.28 (21.40- 36.68)	48.06 (36.11- 64.53)	69.94
Bermuda	0.01 (0.01- 0.01)	0.02 (0.01- 0.02)	100.00	0.01 (0.00- 0.01)	0.01 (0.01- 0.02)	151.30
Bolivia (Plurinational State of)	14498.60 (12803.28- 16303.82)	4993.53 (4540.19- 5483.11)	-65.56	795786.94 (700556.5- 899392.56)	556181.13 (507218.66- 611029.13)	-30.11
Brazil	1463.32 (1240.20- 1711.31)	912.36 (788.20- 1048.06)	-37.65	1918996.9 (1616752.4- 2249374.3)	2164570.3 (1868033.1- 2483588.8)	12.80
Canada	12.62 (11.15- 14.37)	7.29 (6.34- 8.37)	-42.23	3843.56 (3399.36- 4378.21)	3168.60 (2762.52- 3617.78)	-17.56
Chile	3911.59 (3485.31- 4430.37)	1114.17 (987.04- 1261.69)	-71.52	503984.09 (448713.91- 571287.44)	247197.23 (219291.86- 279687.94)	-50.95
Colombia	468.44 (400.30- 545.60)	240.60 (206.44- 280.79)	-48.64	127234.67 (107715.62- 149349.77)	123429.94 (105866.1- 144254.94)	-2.99

Costa Rica	896.06 (744.08- 1069.90)	449.12 (372.40- 526.10)	-49.88	22965.70 (18965.57- 27498.37)	22954.49 (18962.20- 26926.05)	-0.05
Cuba	0.99 (0.86- 1.14)	0.13 (0.09- 0.18)	-86.87	107.13 (92.91- 122.46)	17.78 (12.12- 24.42)	-83.40
Denmark	2.27 (1.97- 2.58)	2.05 (1.72- 2.40)	-9.69	134.88 (117.23- 153.39)	137.31 (114.83- 161.24)	1.80
Dominica	0.02 (0.01- 0.02)	0.57 (0.03- 2.30)	2750.00	0.01 (0.01- 0.01)	0.44 (0.03- 1.78)	4597.84
Dominican Republic	22.03 (19.35- 24.98)	7.02 (6.11- 8.13)	-68.13	1060.87 (930.59- 1206.39)	732.03 (634.09- 847.80)	-31.00
Ecuador	1965.65 (1647.37- 2313.59)	776.74 (653.95- 928.24)	-60.48	167052.69 (139128.86- 197316.98)	132898.13 (111731.95- 159078.03)	-20.45
El Salvador	1292.21 (1104.18- 1516.38)	576.32 (487.87- 670.43)	-55.40	56107.54 (47588.09- 66221.41)	35519.66 (30012.51- 41400.86)	-36.69
France	3.22 (2.86- 3.64)	1.54 (1.34- 1.75)	-52.17	2063.35 (1831.78- 2332.31)	1157.35 (1010.04- 1314.54)	-43.91
Germany	1.27 (1.13- 1.44)	0.65 (0.56- 0.74)	-48.82	1194.84 (1055.05- 1354.01)	647.61 (563.53- 740.64)	-45.80
Grenada	1.94 (1.20- 2.74)	14.35 (8.82- 21.04)	639.69	1.18 (0.75- 1.63)	16.05 (9.90- 23.29)	1259.02
Guatemala	1967.54 (1667.02- 2316.64)	712.89 (605.96- 835.04)	-63.77	121036.18 (101590.44- 143467.08)	110211.03 (92889.24- 129231.16)	-8.94
Guyana	14.46 (11.54- 18.15)	12.15 (9.33- 15.55)	-15.98	78.50 (61.36- 100.52)	85.70 (65.85- 111.84)	9.17
Honduras	1903.46 (1616.01- 2215.56)	904.78 (776.29- 1054.24)	-52.47	66869.28 (56657.88- 78305.21)	76312.81 (65038.65- 88780.77)	14.12
Iceland	0.07 (0.01- 0.15)	0.90 (0.45- 1.73)	1185.71	0.19 (0.03- 0.39)	3.45 (1.67- 6.55)	1725.41
Ireland	0.13 (0.09- 0.18)	3.78 (3.31- 4.30)	2807.69	4.67 (3.22- 6.38)	215.85 (188.25- 246.38)	4525.73
Israel	40.40 (35.63- 45.38)	10.32 (9.06- 11.77)	-74.46	1841.69 (1621.90- 2067.95)	983.44 (864.63- 1119.86)	-46.60
Italy	4.78 (4.23- 5.36)	12.28 (10.87- 14.03)	156.90	3134.50 (2776.68- 3516.82)	8676.86 (7685.51- 9847.33)	176.82
Japan	0.25 (0.22- 0.28)	0.52 (0.45- 0.60)	108.00	358.48 (313.68- 409.86)	751.24 (652.78- 863.21)	109.56
Luxembourg	1.22 (0.89- 1.55)	0.70 (0.30- 1.35)	-42.62	5.51 (4.07- 7.01)	5.35 (2.33- 10.28)	-2.97
Mexico	1347.48 (1120.54- 1614.53)	1030.50 (877.70- 1205.66)	-23.52	939303.44 (769873.56- 1139743.9)	1311109.6 (1115869.6- 1534459.9)	39.58
Netherlands	2.38 (2.10- 2.68)	2.15 (1.86- 2.46)	-9.66	408.78 (361.65- 460.24)	431.16 (374.02- 492.83)	5.48
Nicaragua	1559.28 (1313.79- 1843.13)	668.67 (566.27- 779.08)	-57.12	44839.46 (37587.15- 53213.13)	39908.47 (33606.16- 46667.06)	-11.00

Panama	1025.04 (858.28- 1200.76)	518.47 (434.29- 606.20)	-49.42	21443.64 (17888.40- 25311.50)	21771.47 (18233.92- 25453.20)	1.53
Paraguay	1210.41 (1043.74- 1412.94)	584.81 (500.48- 687.14)	-51.68	40414.16 (34573.66- 47289.16)	39103.23 (33426.53- 46034.36)	-3.24
Peru	1413.44 (1207.69- 1651.78)	635.63 (552.15- 731.03)	-55.03	256753.69 (218829.02- 302078.94)	217437.05 (188880.84- 250655.31)	-15.31
Portugal	5.43 (4.71- 6.20)	5.44 (4.70- 6.28)	0.18	607.82 (528.29- 694.46)	702.11 (609.11- 804.98)	15.51
Puerto Rico	8.53 (7.44- 9.76)	11.76 (10.12- 13.64)	37.87	306.48 (267.45- 350.01)	495.37 (428.09- 571.79)	61.63
Saint Lucia	0.68 (0.37- 1.01)	0.96 (0.22- 2.12)	41.18	0.65 (0.37- 0.96)	2.03 (0.47- 4.46)	211.56
Saint Vincent and the Grenadines	0.09 (0.07- 0.12)	0.15 (0.12- 0.20)	66.67	0.07 (0.05- 0.09)	0.20 (0.15- 0.26)	182.77
Spain	13.62 (12.05- 15.37)	55.76 (49.65- 63.12)	309.40	5783.02 (5115.92- 6522.33)	30863.88 (27428.64- 34841.59)	433.70
Suriname	11.14 (7.68- 15.12)	6.04 (4.31- 7.93)	-45.78	33.81 (22.87- 46.17)	36.10 (25.78- 47.80)	6.76
Sweden	17.72 (15.77- 20.00)	8.59 (7.53- 9.80)	-51.52	1718.83 (1527.38- 1939.41)	1007.12 (880.97- 1148.46)	-41.41
Switzerland	12.34 (10.92- 13.90)	9.49 (8.39- 10.81)	-23.10	1001.75 (885.63- 1131.16)	1015.87 (895.60- 1153.04)	1.41
Trinidad and Tobago	5.40 (4.69- 6.27)	2.06 (1.49- 2.74)	-61.85	52.91 (45.81- 61.25)	34.29 (24.47- 45.91)	-35.19
United Kingdom	0.12 (0.11- 0.14)	0.18 (0.16- 0.21)	50.00	77.61 (68.82- 87.41)	145.66 (127.51- 165.99)	87.68
Uruguay	1529.56 (1289.07- 1792.29)	572.50 (486.99- 669.22)	-62.57	49440.20 (41739.49- 57864.70)	24746.50 (21094.78- 28855.29)	-49.95
Venezuela (Bolivarian Republic of)	2888.71 (2495.06- 3327.43)	1654.73 (1439.50- 1897.81)	-42.72	457479.5 (392331.28- 530739.19)	493902.31 (429738.44- 564220)	7.96
United States of America	19.22 (16.84- 22.10)	16.50 (14.34- 19.04)	-14.15	53505.66 (46913.63- 61492.67)	63553.17 (55365.83- 72997.92)	18.78
Alabama	4.14 (3.59- 4.80)	4.24 (3.66- 4.92)	2.52	187.38 (162.46 - 217.40)	246.44 (213.10 - 285.07)	31.52
Alaska	10.36 (9.15- 11.81)	5.38 (4.71- 6.15)	-48.06	44.95 (39.66 - 51.42)	48.33 (42.33 - 55.27)	7.51
Arizona	9.98 (8.70- 11.51)	5.94 (5.15- 6.87)	-40.49	397.75 (346.89 - 459.10)	497.10 (431.29 - 572.98)	24.98
Arkansas	6.70 (5.80- 7.78)	6.75 (5.81- 7.84)	0.76	175.89 (151.95 - 204.29)	237.74 (205.11 - 275.43)	35.16

California	32.19 (27.95- 37.31)	28.55 (24.68- 33.10)	-11.30	10364.56 (8989.40 - 12023.65)	13600.26 (11761.55 - 15730.08)	31.22
Colorado	8.62 (7.57- 9.90)	5.69 (4.95- 6.56)	-34.03	313.21 (275.08 - 359.43)	364.36 (317.80 - 417.98)	16.33
Connecticut	24.77 (21.86- 28.27)	22.64 (19.87- 25.99)	-8.63	942.72 (832.26 - 1077.58)	983.27 (862.41 - 1121.62)	4.30
Delaware	13.14 (11.46- 15.17)	10.91 (9.45- 12.64)	-16.97	98.56 (85.92 - 113.83)	123.91 (107.46 - 142.84)	25.72
District of Columbia	37.48 (32.65- 43.29)	42.85 (37.20- 49.53)	14.31	266.39 (232.02 - 308.27)	337.05 (291.97 - 388.99)	26.52
Florida	60.67 (53.51- 69.45)	39.16 (34.25- 45.02)	-35.45	8894.82 (7840.39 - 10176.39)	9682.26 (8474.04 - 11081.36)	8.85
Georgia	17.69 (15.49- 20.32)	12.02 (10.44- 13.85)	-32.07	1251.85 (1096.69 - 1439.61)	1502.09 (1307.52 - 1726.49)	19.99
Hawaii	2.65 (2.34- 3.03)	2.02 (1.77- 2.32)	-23.97	32.63 (28.79 - 37.32)	35.00 (30.65 - 40.03)	7.27
Idaho	5.65 (4.94- 6.50)	3.74 (3.25- 4.32)	-33.69	60.20 (52.72 - 69.24)	72.54 (63.12 - 83.38)	20.49
Illinois	7.31 (6.43- 8.38)	6.94 (6.04- 7.99)	-5.10	934.29 (821.34 - 1071.33)	1069.14 (933.22 - 1225.86)	14.43
Indiana	3.56 (3.11- 4.10)	3.51 (3.04- 4.05)	-1.57	220.63 (192.77 - 254.28)	271.74 (236.06 - 312.92)	23.16
Iowa	3.61 (3.15- 4.16)	3.86 (3.35- 4.46)	6.82	111.25 (97.22 - 128.20)	136.74 (118.82 - 157.45)	22.92
Kansas	4.02 (3.51- 4.62)	3.96 (3.44- 4.58)	-1.42	108.97 (95.26 - 125.54)	133.63 (116.15 - 153.84)	22.63
Kentucky	2.81 (2.45- 3.25)	2.82 (2.44- 3.27)	0.54	116.55 (101.58 - 134.80)	148.23 (128.43 - 171.03)	27.19
Louisiana	9.87 (8.53- 11.46)	11.23 (9.68- 13.03)	13.85	445.17 (384.65 - 517.03)	599.41 (517.32 - 694.21)	34.65
Maine	3.17 (2.76- 3.66)	3.61 (3.12- 4.19)	14.08	44.09 (38.40 - 51.03)	56.40 (48.85 - 65.10)	27.92
Maryland	39.31 (34.18- 45.48)	37.65 (32.55- 43.66)	-4.23	2133.10 (1854.11 - 2472.47)	2760.17 (2388.22 - 3188.78)	29.40
Massachusetts	23.95 (21.07- 27.45)	23.23 (20.26- 26.71)	-2.99	1637.90 (1441.21 - 1876.57)	1847.90 (1614.29 - 2117.59)	12.82
Michigan	2.47 (2.17- 2.84)	2.71 (2.36- 3.14)	9.74	255.62 (224.22 - 293.60)	301.52 (262.78 - 346.21)	17.96
Minnesota	7.31 (6.41- 8.39)	6.38 (5.55- 7.36)	-12.68	350.60 (307.62 - 402.60)	411.79 (358.98 - 472.74)	17.45
Mississippi	2.42 (2.11- 2.81)	2.46 (2.13- 2.86)	1.65	66.49 (57.89 - 76.97)	85.19 (73.76 - 98.34)	28.11
Missouri	3.85 (3.36- 4.44)	3.80 (3.30- 4.40)	-1.37	221.25 (193.10 - 255.19)	274.97 (238.69 - 316.80)	24.28

Montana	2.64 (2.31- 3.04)	2.36 (2.05- 2.73)	-10.70	23.40 (20.52 - 26.90)	27.80 (24.22 - 31.93)	18.79
Nebraska	6.68 (5.78- 7.75)	7.12 (6.13- 8.26)	6.56	115.31 (99.67 - 133.91)	154.72 (133.57 - 179.17)	34.17
Nevada	44.31 (38.57- 51.31)	19.51 (16.88- 22.61)	-55.97	576.52 (501.05 - 668.32)	749.09 (648.11 - 865.54)	29.93
New Hampshire	5.82 (5.13- 6.68)	5.08 (4.43- 5.86)	-12.79	72.07 (63.45 - 82.62)	80.26 (70.15 - 91.95)	11.36
New Jersey	45.04 (39.77- 51.48)	40.61 (35.61- 46.61)	-9.82	4041.28 (3565.47 - 4622.57)	4338.88 (3799.74 - 4961.83)	7.36
New Mexico	5.48 (4.80- 6.30)	4.30 (3.73- 4.96)	-21.68	88.77 (77.70 - 102.14)	107.63 (93.63 - 123.74)	21.24
New York	38.32 (33.83- 43.82)	36.11 (31.63- 41.35)	-5.79	7913.06 (6979.19 - 9051.46)	8528.05 (7467.97 - 9755.54)	7.77
North Carolina	13.76 (12.00- 15.89)	10.54 (9.13- 12.21)	-23.40	1036.79 (903.81 - 1198.20)	1309.63 (1135.29 - 1510.47)	26.32
North Dakota	4.24 (3.72- 4.86)	4.17 (3.63- 4.80)	-1.70	28.93 (25.42 - 33.20)	33.42 (29.16 - 38.33)	15.52
Ohio	2.14 (1.88- 2.45)	2.30 (2.00- 2.65)	7.35	260.82 (228.91 - 299.44)	305.77 (266.58 - 350.95)	17.23
Oklahoma	4.42 (3.84- 5.13)	4.50 (3.88- 5.22)	1.80	155.24 (134.58 - 180.12)	204.37 (176.72 - 236.42)	31.65
Oregon	5.38 (4.70- 6.19)	4.39 (3.81- 5.07)	-18.42	172.93 (151.38 - 199.04)	209.35 (182.10 - 240.82)	21.06
Pennsylvania	5.17 (4.55- 5.92)	5.25 (4.58- 6.05)	1.66	701.27 (617.02 - 803.37)	791.78 (691.63 - 907.30)	12.91
Rhode Island	26.15 (22.75- 30.25)	30.46 (26.36- 35.31)	16.48	296.28 (258.03 - 342.89)	378.63 (327.92 - 437.04)	27.80
South Carolina	8.60 (7.55- 9.87)	6.58 (5.72- 7.60)	-23.52	330.77 (290.45 - 379.59)	385.47 (336.17 - 442.24)	16.54
South Dakota	5.38 (4.69- 6.21)	5.18 (4.49- 6.00)	-3.73	39.85 (34.75 - 46.00)	49.88 (43.27 - 57.49)	25.18
Tennessee	6.45 (5.62- 7.46)	5.81 (5.03- 6.73)	-10.02	360.03 (313.75 - 416.45)	458.25 (397.02 - 528.78)	27.28
Texas	24.80 (21.57- 28.72)	17.62 (15.25- 20.42)	-28.96	4468.89 (3884.64 - 5179.59)	5780.83 (5001.98 - 6678.14)	29.36
Utah	22.72 (20.00- 26.03)	11.87 (10.37- 13.61)	-47.75	361.10 (317.89 - 413.86)	400.67 (350.16 - 459.04)	10.96
Vermont	5.48 (4.80- 6.30)	5.64 (4.91- 6.53)	2.89	34.41 (30.17 - 39.55)	40.86 (35.60 - 46.93)	18.74
Virginia	31.04 (27.09- 35.74)	25.85 (22.43- 29.88)	-16.73	2162.02 (1887.05 - 2493.28)	2682.47 (2328.80 - 3090.51)	24.07
Washington	7.01 (6.13- 8.06)	5.39 (4.68- 6.24)	-23.07	380.37 (332.50 - 438.24)	466.57 (405.52 - 537.14)	22.66

West Virginia	1.73 (1.51- 1.99)	1.98 (1.72- 2.29)	14.53	35.55 (31.14 - 40.88)	42.69 (37.16 - 49.08)	20.11
Wisconsin	2.76 (2.42- 3.16)	2.50 (2.18- 2.89)	-9.20	149.07 (131.05 - 170.90)	170.28 (148.65 - 195.23)	14.23
Wyoming	4.96 (4.35- 5.70)	4.09 (3.55- 4.72)	-17.54	24.11 (21.14 - 27.71)	28.64 (24.95 - 32.90)	18.77

Supplementary Table 2. Number of disability-adjusted life-years and age-standardized disability-adjusted life-year rates (per 100,000 population) due to Chagas disease in 1990 and 2019 and the percentage change by country/US state.

Country/State	DALY rate per 100,000 population in 1990	DALY rate per 100,000 population in 2019	Percentage Change 1990-2019 (%)	DALYs (number) 1990	DALYs (number) 2019	Percentage Change 1990-2019 (%)
Andorra	0.11 (0.06 - 0.18)	0.15 (0.09 - 0.26)	34.65	0.07 (0.04 - 0.11)	0.18 (0.10 - 0.31)	154.20
Argentina	116.02 (69.25 - 184.24)	45.77 (28.66 - 119.24)	-60.54	36724.31 (21979.75 - 58618.17)	23552.58 (14648.12 - 61744.26)	-35.87
Australia	0.14 (0.09 - 0.20)	0.04 (0.03 - 0.06)	-69.48	25.55 (16.61 - 37.03)	13.82 (8.74 - 20.32)	-45.92
Austria	0.02 (0.01 - 0.03)	0.01 (0.01 - 0.02)	-45.22	1.91 (1.23 - 2.78)	1.37 (0.84 - 2.01)	-28.33
Belgium	3.86 (2.94 - 4.81)	0.00 (0.00 - 0.00)	-100.00	583.01 (443.89 - 723.71)	0.00 (0.00 - 0.00)	-100.00
Belize	3.68 (1.34 - 5.16)	1.13 (0.71 - 2.58)	-69.29	3.25 (1.22 - 4.52)	2.83 (1.83 - 6.29)	-12.80
Bolivia (Plurinational State of)	339.96 (143.26 - 531.52)	183.76 (64.92 - 328.59)	-45.95	12503.39 (5668.15 - 18939.51)	16882.47 (6156.26 - 29128.09)	35.02
Brazil	245.46 (80.79 - 312.29)	72.32 (44.93 - 125.48)	-70.54	256380.58 (81679.58 - 328720.59)	174194.22 (109039.60 - 302974.38)	-32.06
Canada	0.20 (0.12 - 0.33)	0.07 (0.04 - 0.10)	-66.96	60.26 (36.70 - 96.26)	33.92 (21.43 - 49.37)	-43.71
Chile	40.65 (29.20 - 53.75)	18.01 (12.87 - 24.02)	-55.71	4594.70 (3259.73 - 6165.39)	4195.56 (2987.86 - 5587.42)	-8.69
Colombia	12.26 (9.12 - 21.27)	10.34 (4.79 - 15.53)	-15.67	2617.90 (1966.51 - 4186.43)	5426.21 (2529.02 - 8181.17)	107.27
Costa Rica	9.07 (5.99 - 12.69)	4.59 (2.96 - 6.59)	-49.38	189.64 (123.37 - 270.83)	237.21 (152.93 - 341.43)	25.08
Cuba	0.58 (0.23 - 1.11)	0.00 (0.00 - 0.00)	-99.80	57.16 (22.84 - 109.68)	0.17 (0.09 - 0.29)	-99.70
Denmark	0.02 (0.01 - 0.03)	0.02 (0.01 - 0.03)	-9.83	1.32 (0.85 - 1.94)	1.43 (0.89 - 2.18)	8.47
Dominican Republic	0.26 (0.17 - 0.38)	0.08 (0.05 - 0.11)	-69.79	10.77 (6.91 - 15.67)	7.76 (4.88 - 11.17)	-27.97

Ecuador	24.84 (17.14 - 33.00)	9.75 (6.52 - 14.89)	-60.76	1564.36 (1087.03 - 2095.39)	1530.68 (1023.59 - 2342.76)	-2.15
El Salvador	26.84 (17.48 - 39.51)	12.67 (8.19 - 21.96)	-52.79	918.93 (594.81 - 1293.11)	759.50 (489.69 - 1314.18)	-17.35
Finland	1.37 (0.67 - 2.37)	0.00 (0.00 - 0.00)	-100.00	88.20 (42.83 - 151.93)	0.00 (0.00 - 0.00)	-100.00
France	0.16 (0.06 - 0.30)	0.01 (0.01 - 0.02)	-91.35	121.07 (45.87 - 228.55)	12.22 (7.85 - 17.92)	-89.91
Germany	4.32 (3.97 - 4.69)	0.01 (0.00 - 0.01)	-99.87	5192.56 (4789.89 - 5646.65)	6.83 (4.33 - 10.00)	-99.87
Guatemala	26.62 (18.56 - 35.18)	9.98 (7.11 - 14.11)	-62.50	1168.02 (815.76 - 1589.49)	1251.93 (877.03 - 1753.81)	7.18
Guyana	4.13 (1.37 - 5.99)	1.21 (0.76 - 2.56)	-70.82	11.86 (4.23 - 16.56)	5.95 (3.80 - 12.17)	-49.80
Honduras	24.53 (14.65 - 33.85)	15.80 (7.81 - 24.79)	-35.61	607.24 (384.35 - 843.46)	1003.25 (538.33 - 1518.92)	65.21
Israel	2.65 (1.43 - 4.23)	0.10 (0.06 - 0.15)	-96.20	97.14 (54.82 - 154.80)	10.19 (6.57 - 15.06)	-89.51
Italy	0.04 (0.03 - 0.06)	0.10 (0.07 - 0.15)	137.93	31.33 (20.24 - 45.16)	91.34 (58.99 - 133.67)	191.52
Jamaica	3.26 (1.29 - 6.12)	0.00 (0.00 - 0.00)	-100.00	57.82 (22.85 - 108.70)	0.00 (0.00 - 0.00)	-100.00
Japan	0.00 (0.00 - 0.00)	0.00 (0.00 - 0.01)	83.30	3.64 (2.36 - 5.28)	8.12 (5.16 - 11.87)	122.99
Luxembourg	0.01 (0.01 - 0.02)	0.01 (0.00 - 0.01)	-39.50	0.05 (0.03 - 0.07)	0.05 (0.02 - 0.11)	6.87
Mexico	12.64 (8.35 - 17.93)	10.22 (6.79 - 14.28)	-19.11	6933.23 (4479.72 - 9991.35)	12533.67 (8303.68 - 17462.50)	80.78
Netherlands	0.02 (0.01 - 0.03)	0.02 (0.01 - 0.03)	-14.88	4.09 (2.66 - 5.94)	4.57 (2.92 - 6.66)	11.91
Nicaragua	20.75 (13.90 - 28.09)	9.12 (6.51 - 12.88)	-56.02	411.15 (278.57 - 569.22)	451.35 (316.61 - 645.84)	9.78
Panama	12.78 (8.70 - 16.96)	5.92 (3.78 - 8.69)	-53.63	228.33 (151.79 - 307.84)	247.98 (159.04 - 364.28)	8.60
Paraguay	55.93 (25.82 - 72.47)	26.21 (15.76 - 44.77)	-53.14	1358.92 (659.02 - 1748.67)	1541.27 (947.25 - 2653.88)	13.42
Peru	21.51 (13.69 - 28.98)	8.84 (6.21 - 12.42)	-58.89	2960.96 (1935.81 - 3997.53)	2926.03 (2044.39 - 4115.21)	-1.18
Portugal	0.05 (0.03 - 0.08)	0.05 (0.03 - 0.07)	-8.60	6.29 (3.98 - 9.15)	7.56 (4.76 - 11.25)	20.17
Puerto Rico	3.63 (1.90 - 5.96)	0.11 (0.07 - 0.16)	-97.02	111.07 (58.79 - 181.71)	5.38 (3.40 - 7.94)	-95.15
Spain	0.31 (0.19 - 0.48)	0.48 (0.31 - 0.71)	54.91	157.85 (93.40 - 252.53)	322.37 (206.60 - 469.84)	104.23

Suriname	2.51 (0.98 - 3.57)	0.84 (0.50 - 1.99)	-66.36	6.15 (2.40 - 8.65)	4.45 (2.73 - 10.32)	-27.59
Sweden	0.16 (0.10 - 0.23)	0.08 (0.05 - 0.11)	-50.97	17.10 (11.14 - 24.75)	10.53 (6.66 - 15.31)	-38.40
Switzerland	0.11 (0.07 - 0.16)	0.08 (0.05 - 0.12)	-25.16	9.96 (6.54 - 14.45)	10.61 (6.75 - 15.31)	6.52
Trinidad and Tobago	0.06 (0.04 - 0.09)	0.02 (0.01 - 0.03)	-65.73	0.55 (0.35 - 0.81)	0.37 (0.21 - 0.59)	-32.31
United Kingdom	0.00 (0.00 - 0.00)	0.00 (0.00 - 0.00)	55.76	0.78 (0.50 - 1.12)	1.58 (1.00 - 2.29)	103.27
Uruguay	16.96 (10.66 - 23.34)	7.17 (4.80 - 9.84)	-57.74	587.78 (373.08 - 808.67)	350.72 (237.93 - 484.18)	-40.33
Venezuela (Bolivarian Republic of)	228.02 (99.96 - 285.06)	92.55 (62.26 - 169.48)	-59.41	23314.70 (10840.66 - 28919.13)	27037.40 (18134.19 - 50564.87)	15.97
United States of America	0.21 (0.14 - 0.29)	0.16 (0.10 - 0.23)	-23.84	605.56 (401.39 - 862.61)	686.44 (433.59 - 1008.78)	13.36
Alabama	0.04 (0.03 - 0.06)	0.04 (0.03 - 0.06)	-1.21	1.96 (1.25 - 2.86)	2.69 (1.69 - 3.95)	37.35
Alaska	0.15 (0.09 - 0.21)	0.06 (0.04 - 0.08)	-62.13	0.45 (0.29 - 0.67)	0.52 (0.33 - 0.75)	13.61
Arizona	0.10 (0.06 - 0.15)	0.06 (0.04 - 0.08)	-43.37	4.12 (2.65 - 6.04)	5.39 (3.39 - 7.93)	30.76
Arkansas	0.06 (0.04 - 0.09)	0.06 (0.04 - 0.10)	-0.31	1.84 (1.18 - 2.70)	2.60 (1.63 - 3.82)	40.83
California	0.41 (0.27 - 0.60)	0.28 (0.18 - 0.42)	-31.21	132.05 (86.06 - 191.96)	148.22 (93.01 - 218.22)	12.25
Colorado	0.09 (0.06 - 0.13)	0.05 (0.03 - 0.08)	-38.35	3.21 (2.06 - 4.73)	3.92 (2.48 - 5.76)	22.21
Connecticut	0.24 (0.15 - 0.34)	0.21 (0.13 - 0.31)	-12.46	9.54 (6.15 - 14.06)	10.46 (6.66 - 15.26)	9.65
Delaware	0.13 (0.09 - 0.19)	0.10 (0.06 - 0.15)	-23.24	1.02 (0.66 - 1.50)	1.35 (0.85 - 1.98)	31.81
District of Columbia	0.37 (0.24 - 0.54)	0.44 (0.28 - 0.64)	18.35	2.82 (1.81 - 4.13)	3.67 (2.31 - 5.39)	29.97
Florida	0.59 (0.39 - 0.88)	0.35 (0.22 - 0.52)	-40.22	100.55 (65.48 - 150.45)	104.01 (65.94 - 152.06)	3.44
Georgia	0.18 (0.12 - 0.27)	0.12 (0.08 - 0.18)	-35.28	12.88 (8.27 - 18.94)	16.23 (10.25 - 23.88)	26.03
Hawaii	0.03 (0.02 - 0.04)	0.02 (0.01 - 0.03)	-30.87	0.33 (0.21 - 0.49)	0.37 (0.24 - 0.54)	12.22
Idaho	0.06 (0.04 - 0.08)	0.04 (0.02 - 0.05)	-35.91	0.62 (0.40 - 0.91)	0.78 (0.49 - 1.15)	26.36
Illinois	0.07 (0.05 - 0.10)	0.07 (0.04 - 0.10)	-7.69	9.56 (6.14 - 14.10)	11.49 (7.28 - 16.83)	20.20
Indiana	0.04 (0.02 - 0.05)	0.03 (0.02 - 0.05)	-4.90	2.28 (1.47 - 3.35)	2.94 (1.85 - 4.33)	29.00
Iowa	0.03 (0.02 - 0.05)	0.04 (0.02 - 0.05)	5.47	1.15 (0.74 - 1.69)	1.48 (0.93 - 2.18)	28.82
Kansas	0.04 (0.02 - 0.06)	0.04 (0.02 - 0.06)	-2.53	1.13 (0.72 - 1.65)	1.45 (0.91 - 2.13)	28.45
Kentucky	0.03 (0.02 - 0.04)	0.03 (0.02 - 0.04)	-3.45	1.21 (0.78 - 1.77)	1.61 (1.01 - 2.38)	32.98

Louisiana	0.10 (0.06 - 0.15)	0.11 (0.07 - 0.16)	8.27	4.66 (2.97 - 6.82)	6.56 (4.11 - 9.69)	40.72
Maine	0.03 (0.02 - 0.05)	0.03 (0.02 - 0.05)	4.49	0.46 (0.29 - 0.67)	0.61 (0.39 - 0.90)	33.82
Maryland	0.41 (0.26 - 0.60)	0.36 (0.23 - 0.54)	-10.31	22.07 (14.22 - 32.47)	30.00 (18.87 - 44.16)	35.97
Massachusetts	0.23 (0.15 - 0.34)	0.22 (0.14 - 0.32)	-6.31	16.72 (10.74 - 24.60)	19.84 (12.58 - 29.02)	18.63
Michigan	0.02 (0.02 - 0.04)	0.03 (0.02 - 0.04)	1.78	2.63 (1.69 - 3.87)	3.25 (2.06 - 4.77)	23.75
Minnesota	0.07 (0.05 - 0.10)	0.06 (0.04 - 0.09)	-16.30	3.60 (2.31 - 5.30)	4.44 (2.81 - 6.51)	23.22
Mississippi	0.02 (0.02 - 0.04)	0.02 (0.01 - 0.03)	-1.20	0.69 (0.44 - 1.01)	0.93 (0.58 - 1.37)	34.06
Missouri	0.04 (0.02 - 0.05)	0.04 (0.02 - 0.05)	-3.45	2.29 (1.47 - 3.36)	2.98 (1.88 - 4.39)	30.26
Montana	0.03 (0.02 - 0.04)	0.02 (0.01 - 0.03)	-15.66	0.24 (0.15 - 0.35)	0.30 (0.19 - 0.44)	24.69
Nebraska	0.06 (0.04 - 0.09)	0.07 (0.04 - 0.10)	5.08	1.21 (0.77 - 1.77)	1.69 (1.06 - 2.48)	39.87
Nevada	0.50 (0.33 - 0.74)	0.19 (0.12 - 0.29)	-61.68	5.88 (3.79 - 8.63)	8.12 (5.11 - 11.94)	38.15
New Hampshire	0.06 (0.04 - 0.09)	0.05 (0.03 - 0.07)	-20.84	0.73 (0.47 - 1.08)	0.86 (0.55 - 1.26)	17.08
New Jersey	0.43 (0.28 - 0.63)	0.38 (0.24 - 0.56)	-12.51	40.82 (26.30 - 59.84)	46.27 (29.48 - 67.58)	13.35
New Mexico	0.06 (0.04 - 0.08)	0.04 (0.03 - 0.06)	-28.15	0.92 (0.59 - 1.35)	1.16 (0.73 - 1.71)	27.14
New York	0.45 (0.29 - 0.64)	0.34 (0.21 - 0.50)	-24.15	102.27 (66.13 - 147.58)	91.26 (58.03 - 133.25)	-10.76
North Carolina	0.14 (0.09 - 0.20)	0.10 (0.06 - 0.15)	-27.25	10.75 (6.90 - 15.78)	14.22 (8.94 - 20.95)	32.32
North Dakota	0.04 (0.03 - 0.06)	0.04 (0.02 - 0.06)	-3.47	0.30 (0.19 - 0.44)	0.36 (0.23 - 0.53)	21.22
Ohio	0.02 (0.01 - 0.03)	0.02 (0.01 - 0.03)	2.22	2.68 (1.72 - 3.94)	3.29 (2.08 - 4.83)	22.99
Oklahoma	0.04 (0.03 - 0.06)	0.04 (0.03 - 0.06)	0.37	1.62 (1.04 - 2.37)	2.23 (1.40 - 3.28)	37.53
Oregon	0.05 (0.03 - 0.08)	0.04 (0.03 - 0.06)	-21.77	1.78 (1.15 - 2.62)	2.26 (1.43 - 3.33)	26.93
Pennsylvania	0.05 (0.03 - 0.07)	0.05 (0.03 - 0.07)	-0.74	7.17 (4.60 - 10.58)	8.50 (5.39 - 12.43)	18.56
Rhode Island	0.25 (0.16 - 0.37)	0.29 (0.18 - 0.42)	13.16	3.08 (1.97 - 4.51)	4.12 (2.59 - 6.06)	33.71
South Carolina	0.09 (0.06 - 0.13)	0.06 (0.04 - 0.09)	-29.10	3.39 (2.18 - 5.00)	4.15 (2.63 - 6.10)	22.52
South Dakota	0.05 (0.03 - 0.07)	0.05 (0.03 - 0.07)	-5.39	0.41 (0.27 - 0.61)	0.54 (0.34 - 0.80)	31.07
Tennessee	0.06 (0.04 - 0.09)	0.06 (0.03 - 0.08)	-13.55	3.74 (2.40 - 5.48)	4.98 (3.13 - 7.34)	33.32
Texas	0.26 (0.17 - 0.38)	0.18 (0.11 - 0.26)	-31.07	46.41 (29.79 - 67.88)	62.89 (39.59 - 92.22)	35.51
Utah	0.24 (0.15 - 0.35)	0.12 (0.08 - 0.18)	-48.43	3.67 (2.36 - 5.40)	4.29 (2.73 - 6.27)	16.84
Vermont	0.05 (0.04 - 0.08)	0.05 (0.03 - 0.08)	-6.92	0.35 (0.23 - 0.52)	0.44 (0.28 - 0.65)	24.53
Virginia	0.32 (0.20 - 0.47)	0.25 (0.16 - 0.37)	-21.73	22.22 (14.25 - 32.55)	29.04 (18.30 - 42.72)	30.70
Washington	0.07 (0.05 - 0.10)	0.05 (0.03 - 0.08)	-27.13	3.93 (2.53 - 5.77)	5.05 (3.18 - 7.44)	28.61

West Virginia	0.02 (0.01 - 0.02)	0.02 (0.01 - 0.03)	10.19	0.37 (0.24 - 0.54)	0.46 (0.29 - 0.68)	25.95
Wisconsin	0.03 (0.02 - 0.04)	0.02 (0.01 - 0.03)	-12.71	1.53 (0.98 - 2.25)	1.83 (1.16 - 2.68)	19.93
Wyoming	0.05 (0.03 - 0.07)	0.04 (0.02 - 0.06)	-23.09	0.25 (0.16 - 0.37)	0.31 (0.20 - 0.45)	24.62

Graphical Abstract

Click here to access/download;Figure;Graphical_Abstract.jpg

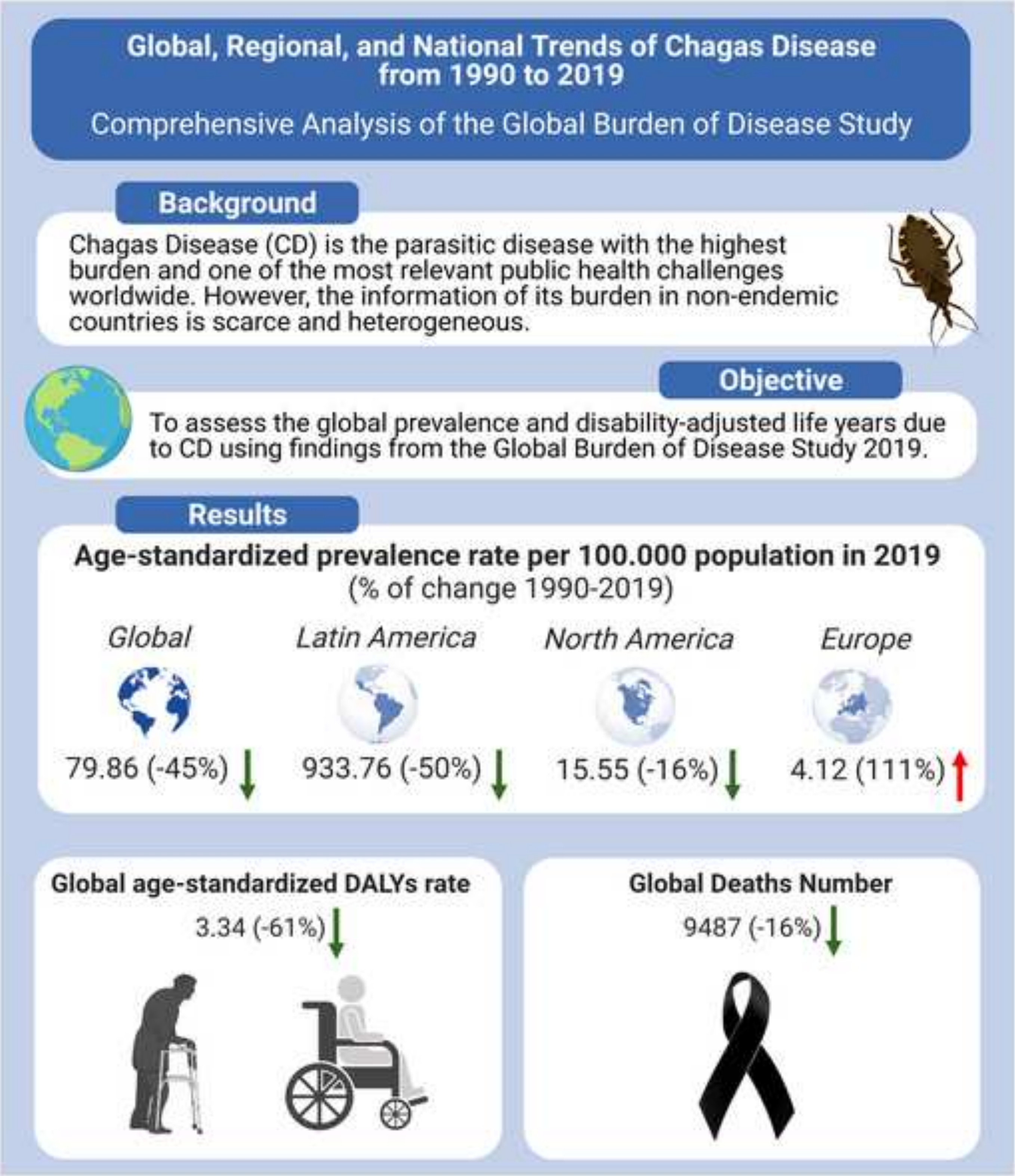

Supplement: Supplementary File. — Supplementary figures 1 to 13 and Tables 1 and 2. [file gh-17-1-1150-s1.pdf]
